# TIAM1 drives prostatic branching phenotype and is a potential therapeutic target for benign prostatic hyperplasia

**DOI:** 10.1172/jci.insight.188062

**Published:** 2025-05-20

**Authors:** Hamed Khedmatgozar, Sayanika Dutta, Michael Dominguez, Murugananthkumar Raju, Girijesh Kumar Patel, Daniel Latour, Melanie K. Johnson, Mohamed Fokar, Irfan Warraich, Allan Haynes, Barry J. Maurer, Werner de Riese, Luis Brandi, Robert J. Matusik, Srinivas Nandana, Manisha Tripathi

**Affiliations:** 1 Department of Cell Biology and Biochemistry, Texas Tech University Health Sciences Center (TTUHSC), Lubbock, Texas, USA.; 2 Center for Biotechnology and Biochemistry, Texas Tech University, Lubbock, Texas, USA.; 3 Department of Pathology,; 4 Department of Urology,; 5 Department of Pediatrics, and; 6 Department of Medicine, TTUHSC, Lubbock, Texas, USA.; 7 Department of Urology, Vanderbilt University Medical Center, Nashville, Tennessee, USA.

**Keywords:** Aging, Cell biology, Urology

## Abstract

Benign prostatic hyperplasia (BPH) is the most common urologic condition in elderly men, characterized by the reactivation of developmental programs such as prostatic budding and branching. However, the molecular mechanisms underlying this reactivation in BPH remain unclear. In this study, we identified T-lymphoma invasion and metastasis-inducing protein-1 (TIAM1) as a critical regulator of prostatic budding and branching. By generating an unbiased BPH transcriptomic signature from patient datasets, we discovered an upregulation of *TIAM1*, which was subsequently validated at the protein level. Functional assays using organoid cultures derived from human prostatic cell lines revealed that TIAM1 is essential for prostatic budding and branching. Additionally, the BPH transcriptomic signature identified NSC23766, a small molecule inhibitor of TIAM1/RAC1 signaling, as a therapeutic proof-of-concept agent for BPH. Genetic knockdown of *TIAM1* in human prostatic cell lines markedly reduced organoid branching, an effect mirrored by administration of NSC23766. The translational relevance of these findings is underscored by the growth inhibition observed in patient-derived BPH organoids treated with NSC23766. In conclusion, our findings identify TIAM1 as a key driver of prostatic branching and growth, and they suggest that targeting TIAM1/RAC1 signaling could be a promising therapeutic strategy for BPH.

## Abstract 2

TIAM1 is a driver of the prostatic branching phenotype and a potential therapeutic target for benign prostatic hyperplasia

## Graphical abstract

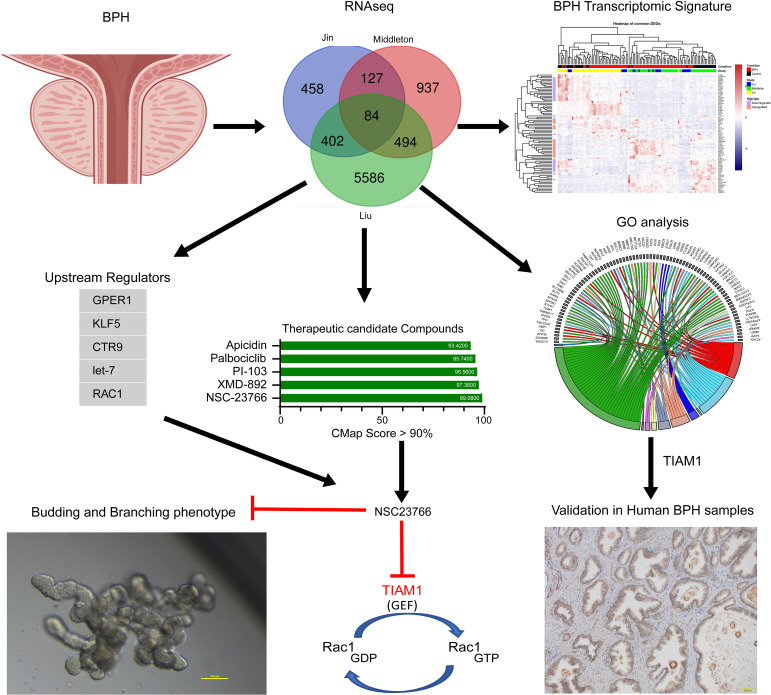

## Introduction

Benign prostatic hyperplasia (BPH) is a common pathological condition that affects more than half of men in their sixth decade and > 90% over 90 years of age (1, 2). BPH causes significant morbidity by obstructing urine outflow (also called bladder outlet obstruction) resulting in lower urinary tract symptoms (LUTS) (1–4), the defining characteristics of BPH. The widespread prevalence of BPH is a significant US healthcare burden currently estimated at $4 billion per annum with costs expected to increase over the next 2 decades due to population demographics (4–9). Characteristic histopathological features of BPH include hyperproliferation of the epithelial and stromal prostate compartments resulting in the enlargement of the gland (10, 11). Currently, the 2 main nonsurgical treatment options are (a) decreasing prostate size by blocking the conversion of testosterone (T) to dihydrotestosterone (DHT) using a 5α-reductase inhibitor (5ARI) and/or (b) reducing symptoms by relaxing smooth muscle tone within the prostate and bladder neck by using an α-adrenergic receptor blocker (α-blocker). However, as the Medical Therapy of Prostatic Symptoms (MTOPS) clinical study reported, these treatment options often fail, leaving surgical correction as the only remaining option (12, 13). Therefore, there is a clear need to identify and develop molecular-based approaches for the treatment of the disease.

Resurgent branching morphogenesis, a key process in prostatic development, has been postulated to lead to the hyperplastic phenotype in BPH (14–17). However, the precise molecular triggers that lead to reactivation of branching morphogenesis in BPH remain elusive. Defining molecular mechanisms responsible for branching phenotypes could, in turn, lead to a better understanding of the causative factors of BPH pathogenesis and new and effective therapeutic strategies for the treatment of the disease.

In this study, using clinical BPH samples and 2D/3D cell culture models, we have utilized a combination of bioinformatic-based analyses, genetic-modulation studies, and a proof-of-concept pharmacological approach to identify T-lymphoma invasion and metastasis-inducing protein-1 (TIAM1), an activator of small GTPase RAC1 signaling, as (a) a driver of branching phenotype in BPH and (b) a promising candidate pathway for molecular approaches for therapeutic targeting of BPH.

## Results

### Identification of a BPH transcriptomic signature.

**Figure 1:**
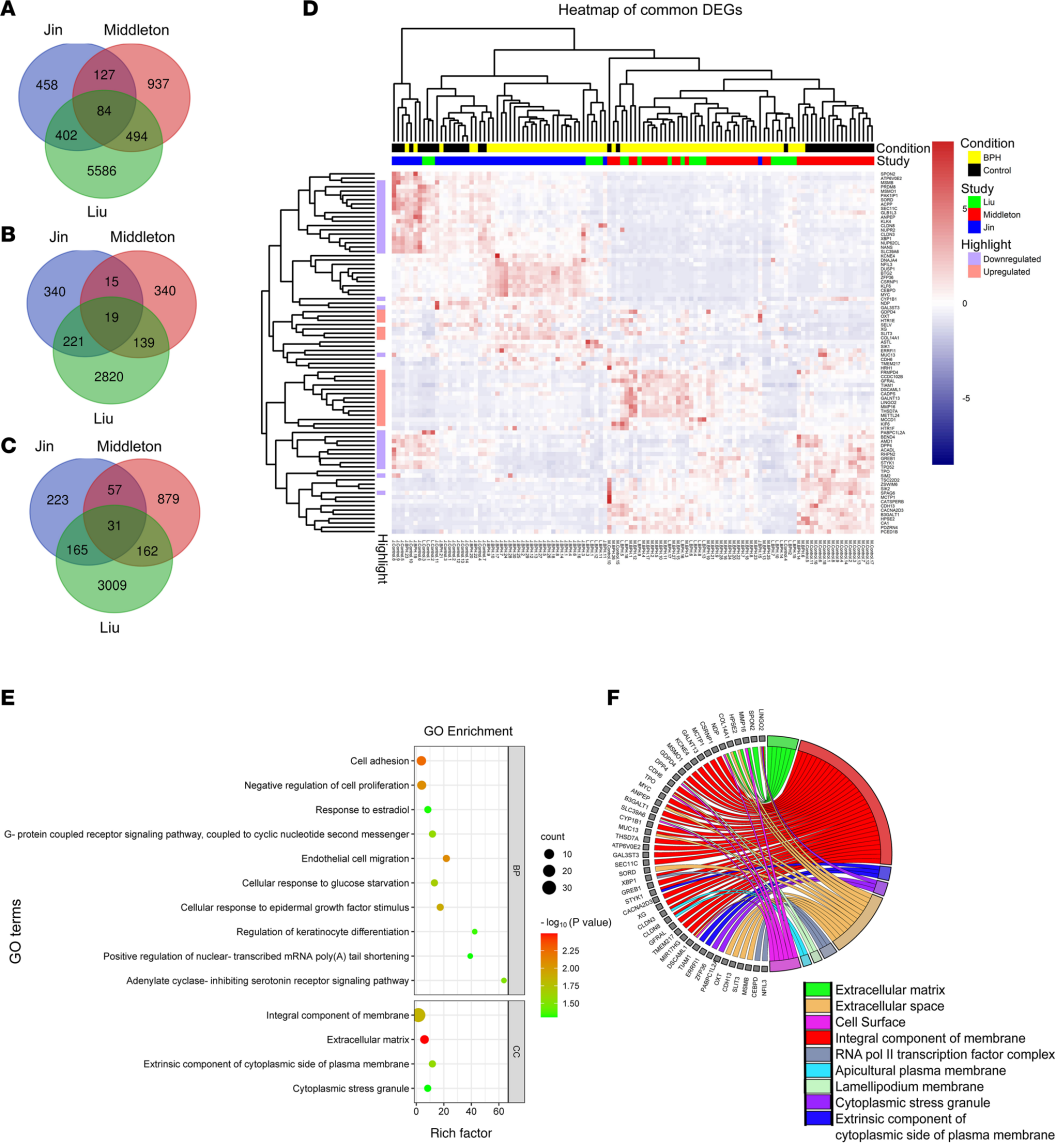
Identification of a BPH transcriptomic signature. (**A**) Venn diagram showing 84 common differentially expressed genes (cDEGs) based on the overlap of individual sets of DEGs obtained from RNA-Seq datasets from 3 independent BPH patient cohorts (Liu et al., Middleton et al., and Jin et al.; refs. 44–46). Differentially expressed genes (DEGs) were filtered with a fold-change of ≥ |1.5| and FDR of < 0.05. (**B**) Venn diagram showing 19 upregulated cDEGs from the 3 datasets. (**C**) Venn diagram showing 31 downregulated cDEGs from the 3 datasets. (**D**) Heatmap representing the normalized patient-wise expression pattern of the cDEGs across the 3 datasets: Jin (blue), Middleton (red), and Liu (green); BPH samples are indicated in yellow, and control samples in black. DEGs upregulated in all 3 datasets are shown in light red, while DEGs downregulated in all 3 datasets are shown in light blue. (**E**) Gene ontology (GO) pathway analysis of the 84 cDEGs, categorized into 2 functional groups: biological processes (BP) and cellular compartments (CC) (*P* < 0.05). (**F**) Circos plot illustrating the correlation between cellular compartments and their associated cDEGs.

In the current study, mRNA expression profiles from 3 independent BPH patient datasets were utilized to compare mRNA expression between BPH and control tissues. Through comparing the RNA-Seq read counts of various genes followed by the application of the cutoff criteria (fold-change ≥ |1.5| and FDR < 0.05), differentially expressed genes (DEGs) were identified for each dataset. Next, the DEGs obtained from each of the 3 datasets were overlapped to arrive at 84 common DEGs (cDEGs) that constituted the putative BPH transcriptomic signature (Figure 1A). The cDEGs included 19 cDEGs consistently upregulated among all 3 datasets (Figure 1B) and 31 cDEGs consistently downregulated in all 3 datasets (Figure 1C). The remaining 34 cDEGs were significantly dysregulated but not consistently up- or downregulated in all 3 datasets. A heatmap of the 84 cDEGs was created (Figure 1D). To garner insights regarding the possible biological functions of the cDEGs, gene enrichment analysis was performed using Database for Annotation, Visualization and Integrated Discovery (DAVID), which included gene ontology (GO) (Figure 1E). For GO biological processes (BP), cell adhesion, negative regulation of cell proliferation, response to estradiol, differentiation, and migration were implicated (Supplemental Figure 1A; supplemental material available online with this article; https://doi.org/10.1172/jci.insight.188062DS1). For the GO cellular component (CC), gene enrichment primarily involved membrane and extracellular matrix (Figure 1F). Functional implications of the cDEGs were further investigated by Reactome pathway analysis. A number of cDEGs were enriched in 6 Reactome pathways, including glycosylation of proteins and cell-cell communication (Supplemental Figure 1B). Taken together, a BPH transcriptomic signature was identified that is associated with key biological BPH processes, including cell-cell communication, cellular proliferation, and adhesion.

### TIAM1/RAC1 signaling as a potential therapeutic target for BPH.

Gene expression data and transcriptomic signatures (Supplemental Figure 1C) have frequently been used as diagnostic and prognostic markers of disease. However, gene expression signatures often include several passenger genes that may not represent disease drivers. On the other hand, upstream regulators often represent crucial factors that drive progression and disease etiology and are better candidates for potential therapeutic interventions. Therefore, QIAGEN Ingenuity Pathway Analysis (IPA) was performed to identify key upstream regulators of the 84 cDEGs. The top 5 upstream regulators (*P* < 0.05) are listed in Table 1 and include G protein-coupled estrogen receptor 1 (GPER1), KLF transcription factor 5 (KLF5), CTR9 Homolog, Paf1/RNA Polymerase II Complex Component (CTR9), *mir-let7* (*let-7*), and Rac family small GTPase 1 (RAC1).

**Figure 2:**
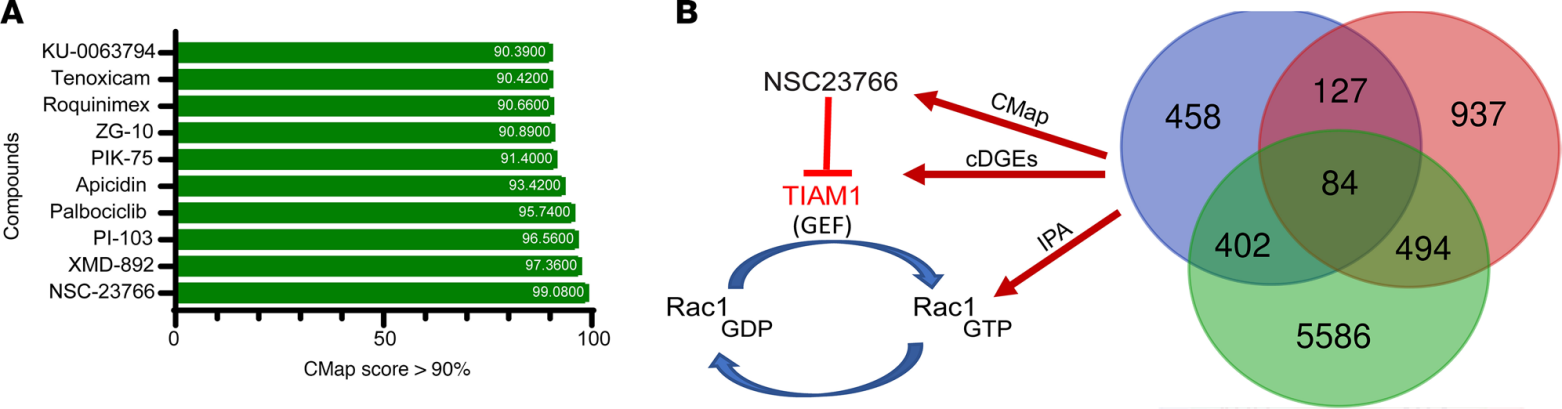
Identification of the upstream regulators and the top therapeutic candidate compound affecting the BPH transcriptomic signature. (**A**) Connectivity Map (CMap) analysis displaying the CMap scores of the top 10 nominated compounds based on the 84 cDEGs. (**B**) A schematic showing the connecting links between: (a) *TIAM1* (a RAC1-specific GEF) as one of the upregulated cDEGs, (b) RAC1 as an upstream regulator based on the 84 cDEGs, and (c) NSC23766, a small molecule inhibitor of TIAM1/RAC1 signaling, as the top candidate compound from the CMap analysis.

The cDEGs were queried to discover potential pathway inhibitors using Connectivity Map (CMap) (L1000 platform; https://clue.io/l1000-query) (18), a widely used in silico drug screening tool that uses cellular responses to perturbation to illustrate relationships between genes, diseases, and therapeutic compounds through CMap score. CMap score is a measure of the degree of correlation with the gene expression signature. Using CMap analysis, the CMap scores were calculated for both upregulated and downregulated cDEGs and therapeutic compounds; a CMap score of 90 or higher was shortlisted as potentially affecting key aspects of the BPH signature (Figure 2A and Supplemental Table 4). Interestingly, the shortlisted compounds included inhibitors of inflammation, fibrosis, and proliferation — the hallmarks of BPH. CMap analysis revealed NSC23766, a small molecule inhibitor of TIAM1 pathway, as the highest ranked candidate compound with a CMap score of 99.08% (Figure 2A). NSC23766 is a small molecule that blocks activation of RAC1 protein by inhibiting TIAM1 activity (19, 20) (Figure 2B); the list of upstream cDEG regulators included the GTPase, *RAC1* (Table 1). Furthermore, analysis of publicly available single-cell RNA-Seq data from BPH and normal prostates revealed expression of *TIAM1* in different cell populations (https://strandlab.net/sc.data/) (21) (Supplemental Figure 1, D–F).

### TIAM1 protein expression is elevated in BPH.

**Figure 3:**
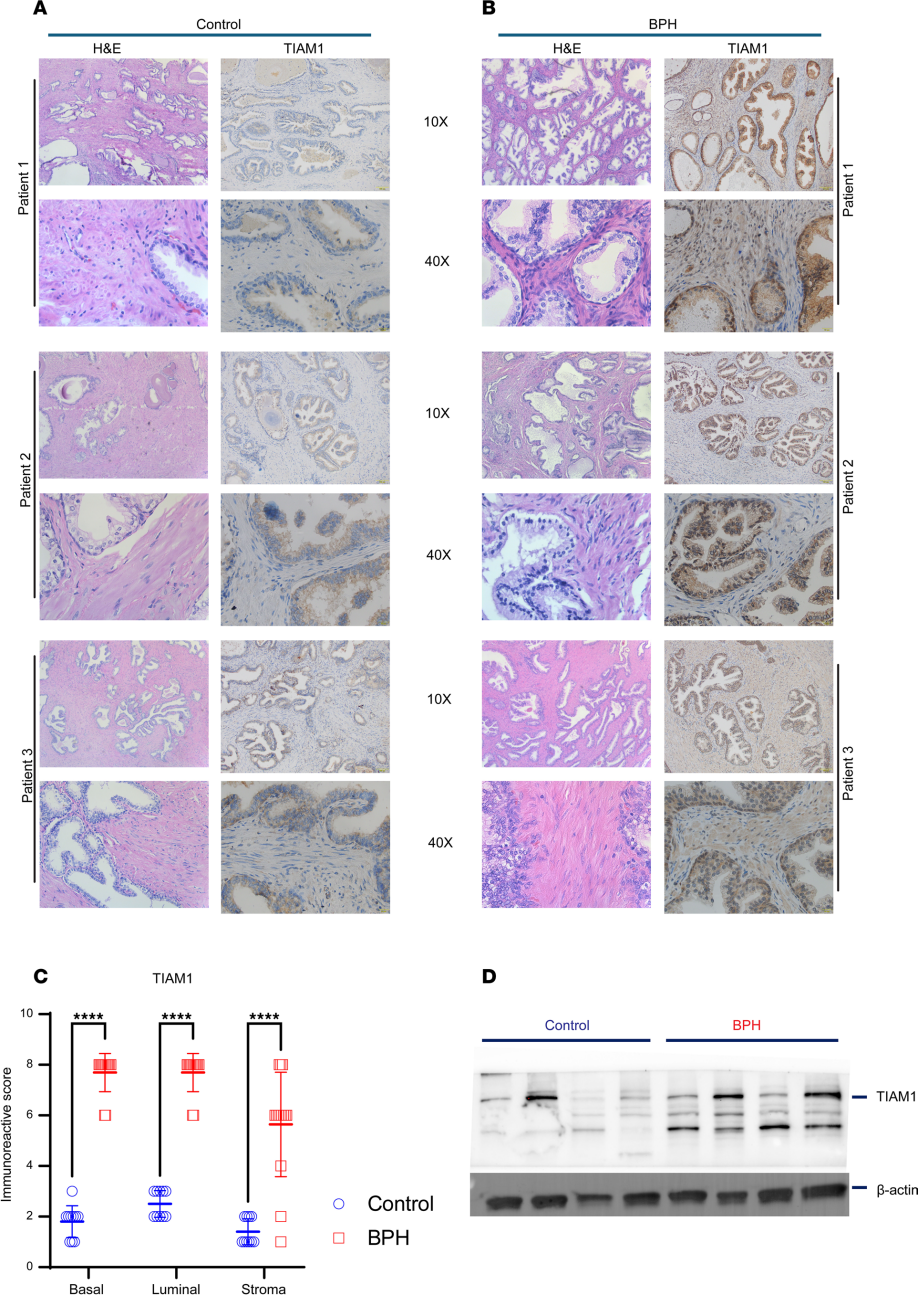
TIAM1 protein expression is elevated in BPH. (**A** and **B**) Representative IHC and H&E staining showing TIAM1 expression at 10× and 40× original magnification in control (**A**) and BPH tissues (**B**). (**C**) Quantification of the IHC images using the immunoreactive score (IRS) in BPH (*n* = 14) compared with control (*n* = 10). The IRS = % of cells × intensity of staining. Two-way ANOVA, **** *P* < 0.0001. (**D**) Western blot analysis showed the expression of TIAM1 in BPH (*n* = 4) compared with control (*n* = 4).

Since (a) NSC23766 is a small molecule inhibitor of TIAM1 activity (20), (b) IPA and CMap analysis supported TIAM1/RAC1 signaling as a possible pathway useful for the therapeutic targeting of BPH (Figure 2B), and (c) the BPH transcriptomic signature revealed TIAM1 upregulation in all 3 datasets, we sought to validate the increase of expression of TIAM1 in a fourth independent patient cohort. Therefore, Cohort 4 specimens were probed for TIAM1 protein by IHC (Figure 3, A–C, and Supplemental Figure 2). Cohort 4 BPH specimens were transition zone (TZ) tissues from simple prostatectomies. Control specimens were benign TZ tissues from radical prostatectomy for low-volume prostate cancer localized to the peripheral zone (PZ). Results revealed a significant increase in TIAM1 protein in BPH tissues compared with control tissues, *P* < 0.0001, in epithelial (basal and luminal compartments) and stromal cells (Figure 3C). Furthermore, Western blot analysis, as an additional detection method, revealed higher TIAM1 protein levels in BPH samples compared with control samples (Figure 3D). Together, the differential increase of TIAM1 protein compared with control tissues was consistent with the differential increase of mRNA levels measured in transcriptome Cohorts 1–3 of patients with BPH (Figure 1D).

### TIAM1 is critical to the prostatic branching phenotype.

**Figure 4:**
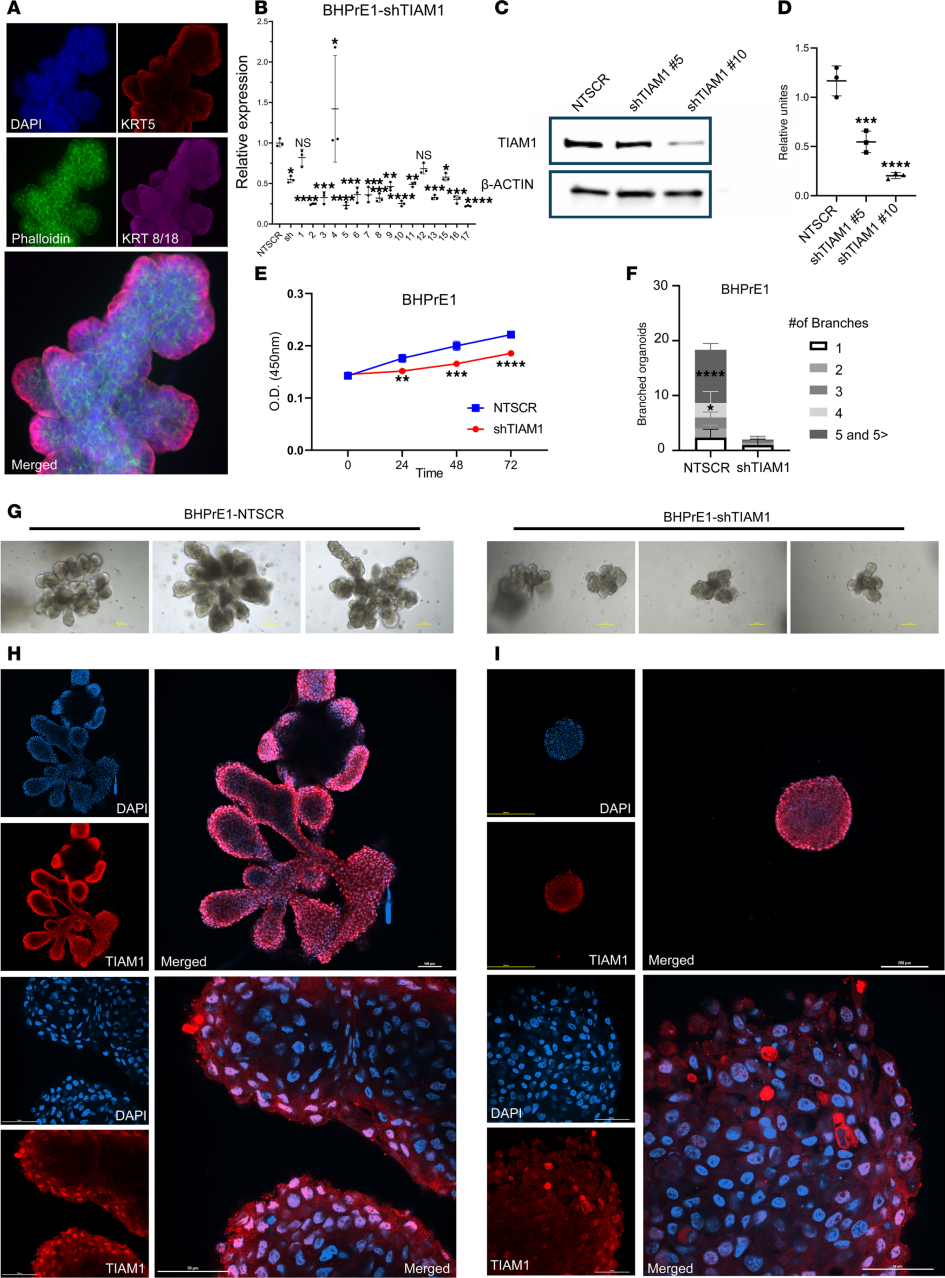
TIAM1 is critical to the prostatic branching phenotype. (**A**) Whole-mount IF staining of BHPrE1 organoids with basal cell marker (KRT5) and luminal cell marker (KRT8/18) on day 9 of culture. Original magnification, 20×. (**B**) qPCR analysis showing decreased mRNA expression of TIAM1 in BHPrE1^shTIAM1^ polyclonal (sh) and monoclonal colonies (indicated as 1–17) compared with BHPrE1^NTSCR^ control cells; 1-way ANOVA was used. (**C** and **D**) Western blot and quantitative analysis showing decreased TIAM1 protein expression in BHPrE1^shTIAM1^ colonies 5 and 10 compared with BHPrE1^NTSCR^ control cells. Each experiment was performed in 3 independent replicates; 1-way ANOVA was used. (**E**) WST-1 assay-based cell proliferation analysis showing a decrease in proliferation in BHPrE1^shTIAM1^ compared with BHPrE1 ^NTSCR^ control cells. *n* = 5; data are shown as mean ± SD. Unpaired 2-tailed Student’s *t* test was performed. **P < 0.01; ***P < 0.001; ****P < 0.0001. (**F**) Quantification of the number of branched organoids on day 12 formed from BHPrE1^shTIAM1^ cells compared with BHPrE1^NTSCR^ control cells. *n* = 3, data are shown as mean ± SD. One-way ANOVA was performed. *P < 0.05; ****P < 0.0001. Each experiment was performed in 3 independent replicates. (**G**) Representative images taken on day 12 of organoids formed from BHPrE1^shTIAM1^ cells compared with organoids from BHPrE1^NTSCR^ Scale bar: 100 μm. (**H**) Whole-mount IF staining of organoids from BHPrE1^NTSCR^ cells stained for TIAM1 (red) and DAPI (blue) on day 15 at 10× and 60× original magnification. The top panel (3 images) was captured at 10× magnification. The bottom panel (3 images) was captured at 60× magnification. Scale bar: 50 μm and 100 μm. (**I**) Whole-mount IF staining of organoids formed from BHPrE1^shTIAM1^ ^#10^ cells stained for TIAM1 (red) and DAPI (blue) on day 15 at 10× and 60× original magnification. The top panel (3 images) was captured at 10× magnification. The bottom panel (3 images) was captured at 60× magnification. Scale bar: 50 μm and 100 μm.

TIAM1 is reported to mediate branching in other organ systems, including neurite outgrowth and angiogenesis (22). The prevailing hypothesis has been that the hyperplastic phenotype in BPH is driven by the reactivation of embryonic branching morphogenesis (14), a developmental program active in the ontogeny of the prostate and other organs. We utilized a 3D organoid culture of human prostatic cells in vitro to model normal embryonic prostatic branching morphogenesis and to investigate the role of TIAM1 in the prostatic branching phenotype. Results demonstrated that BHPrE1 human benign prostatic cells spontaneously organized into KRT5^+^ basal and KRT8/18^+^ luminal layers and eventually budded and branched to form in vivo–like acinar structures (16, 23) as shown by whole-mount immunofluorescence (IF) imaging (Figure 4A).

To functionally characterize the role of TIAM1 in prostatic biology, we performed shRNA knockdown of *TIAM1* in BHPrE1 (BHPrE1^shTIAM1^) cells, and we isolated single clones via serial dilution. We confirmed the knockdown by quantitative PCR (qPCR), protein immunoblotting, and immunocytochemical analyses (Figure 4, B–D, and Supplemental Figure 3). Cell proliferation analysis revealed a significant reduction in the growth of BHPrE1^shTIAM1^ cells compared with the control BHPrE1 nontargeting scrambled control RNA (BHPrE1^NTSCR^) cells (Figure 4E). Notably, organoids formed from BHPrE1^shTIAM1^ cells showed a reduction in both total number of branched organoids and organoids with 4 or more branches (Figure 4, F and G, and Supplemental Figure 4) when compared with controls. The protein expression of TIAM1 in organoids formed from BHPrE1^NTSCR^ and BHPrE1^shTIAM1^ cells was visualized using IF staining (Figure 4, H and I). Next, as a complementary approach, we evaluated the functional effects of *TIAM1* overexpression (TIAM1OE) on prostatic organoid formation. Western blot analysis verified a 2.1-fold increase in TIAM1 protein levels in BHPrE1^TIAM1OE^ cells compared with control cells (Supplemental Figure 5A). Cell proliferation assays revealed that TIAM1OE significantly (*P* < 0.001) enhanced the growth of BHPrE1 cells (Supplemental Figure 5B). Notably, organoids derived from BHPrE1^TIAM1OE^ cells displayed an increase in organoid size and the total number of branched organoids (Supplemental Figure 5, C–G) compared with controls. Additionally, transient transfection of full-length *TIAM1* (TIAM1-FL) in BHPrE1 cells resulted in similar increases in cell proliferation and organoid size compared with controls (Supplemental Figure 6). Thus, genetic modulation experiments using both knockdown and overexpression approaches validated that TIAM1 is associated with increased proliferation, organoid size, and branching phenotype in the BHPrE1 cell model.

### Genetic knockdown of TIAM1 leads to reduced levels of active RAC1 (GTP-RAC1).

TIAM1 functions as a guanine nucleotide exchange factor (GEF) to activate the small GTPase RAC1. To assess the effect of *TIAM1* knockdown on the activation of RAC1, a GST-PAK1-PBD pull-down assay followed by immunoblotting was performed. As expected from its reported function in other cell types, *TIAM1* knockdown decreased the levels of GTP-RAC1 (active RAC1) in BHPrE1^shTIAM1^ cells when compared with BHPrE1^NTSCR^ control cells; however, the levels of total RAC1 remained unaltered (Supplemental Figure 7A).

### NSC23766 exposure resulted in decreased proliferation of human benign prostatic epithelial and stromal cells.

**Figure 5:**
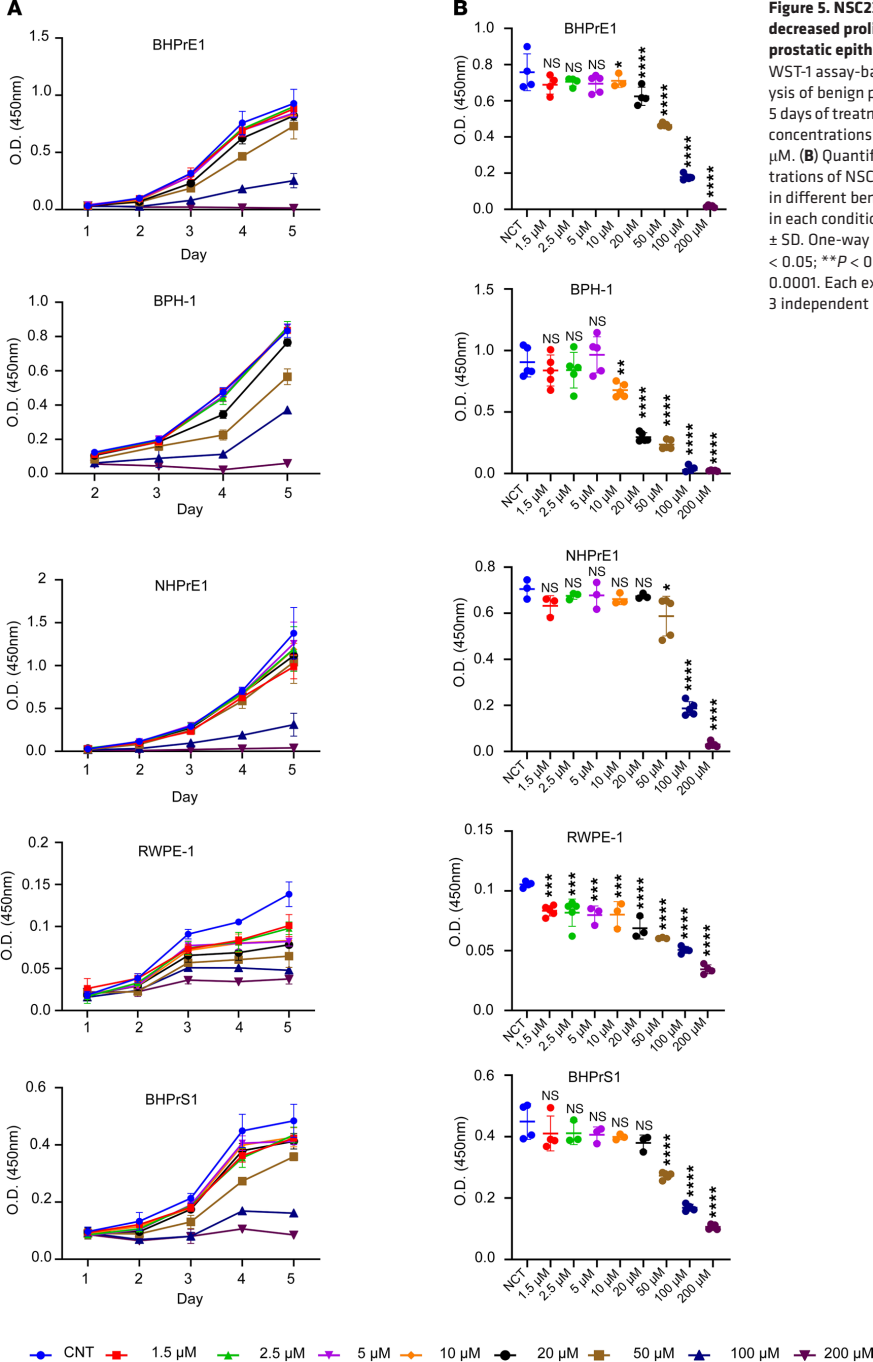
NSC23766 exposure resulted in decreased proliferation of human benign prostatic epithelial and stromal cells. (**A**) WST-1 assay-based cell proliferation analysis of benign prostate cell lines, following 5 days of treatment with NSC23766 at concentrations ranging from 1.25 μM to 200 μM. (**B**) Quantification of different concentrations of NSC23766 treatment on day 4 in different benign prostate cell lines. *n* = 5 in each condition; data are shown as mean ± SD. One-way ANOVA was performed. *P < 0.05; **P < 0.01; ***P < 0.001; ****P < 0.0001. Each experiment was performed in 3 independent replicates.

Increased proliferation of both epithelial and stromal compartments is a defining feature of BPH. NSC23766, a commonly utilized small molecule RAC1 inhibitor, is known for its ability to hinder RAC1 activation via the RAC1-specific GEFs, particularly TIAM1. To assess whether pharmacological inhibition of TIAM1 using NSC23766 could impair proliferation of prostatic cells, we treated benign human prostate epithelial cells (BHPrE1), nontumorigenic human prostate epithelial cells (BPH-1), normal human prostate epithelial cells (NHPrE1), normal human prostate epithelial cells (RWPE-1), and benign human prostate stromal cells (BHPrS1) with NSC23766 concentrations ranging from 1.25 μM to 200 μM. Exposure of NSC23766 caused a significant decrease in proliferation across the benign prostatic epithelial and stromal cell lines in a dose-dependent manner (Figure 5 and Supplemental Figure 8). The half-maximal inhibitory concentrations (IC_50_) of NSC23766 for BHPrE1, NHPrE1, BPH-1, RWPE-1, and BHPrS1 cells were observed at 48.3 μM, 52.3 μM, 38.4 μM, 10.3 μM, and 33.0 μM, respectively (Supplemental Figure 8). In addition, as with genetic knockdown of *TIAM1*, NSC23766-mediated pharmacological inhibition of TIAM1 in BHPrE1 cells led to a decrease in active RAC1 levels while not affecting total RAC1 (Supplemental Figure 7B). To evaluate the effect of NSC23766 on cellular apoptosis, we used an annexin V and propidium iodide (PI) staining assay on human benign prostate cell lines BHPrE1, NHPrE1, and BHPrS1. After 48 hours of treatment with NSC23766 (10 μM), the percentage of live cells in each cell line was assessed and compared with vehicle control cells using flow cytometric analysis. Results indicate that NSC23766 did not induce significant changes in the percentage of live cells in any of the tested cell lines. Furthermore, we performed the annexin V/ PI staining on BHPrE1 cells cultured in both 2D and 3D models. Both models displayed comparable levels of live cells between the treated and control groups, suggesting that NSC23766 (10 μM) did not significantly increase baseline apoptosis under these conditions (Supplemental Figure 9).

### NSC23766 induced an impairment of prostatic organoid branching that phenocopied genetic knockdown of TIAM1.

**Figure 6:**
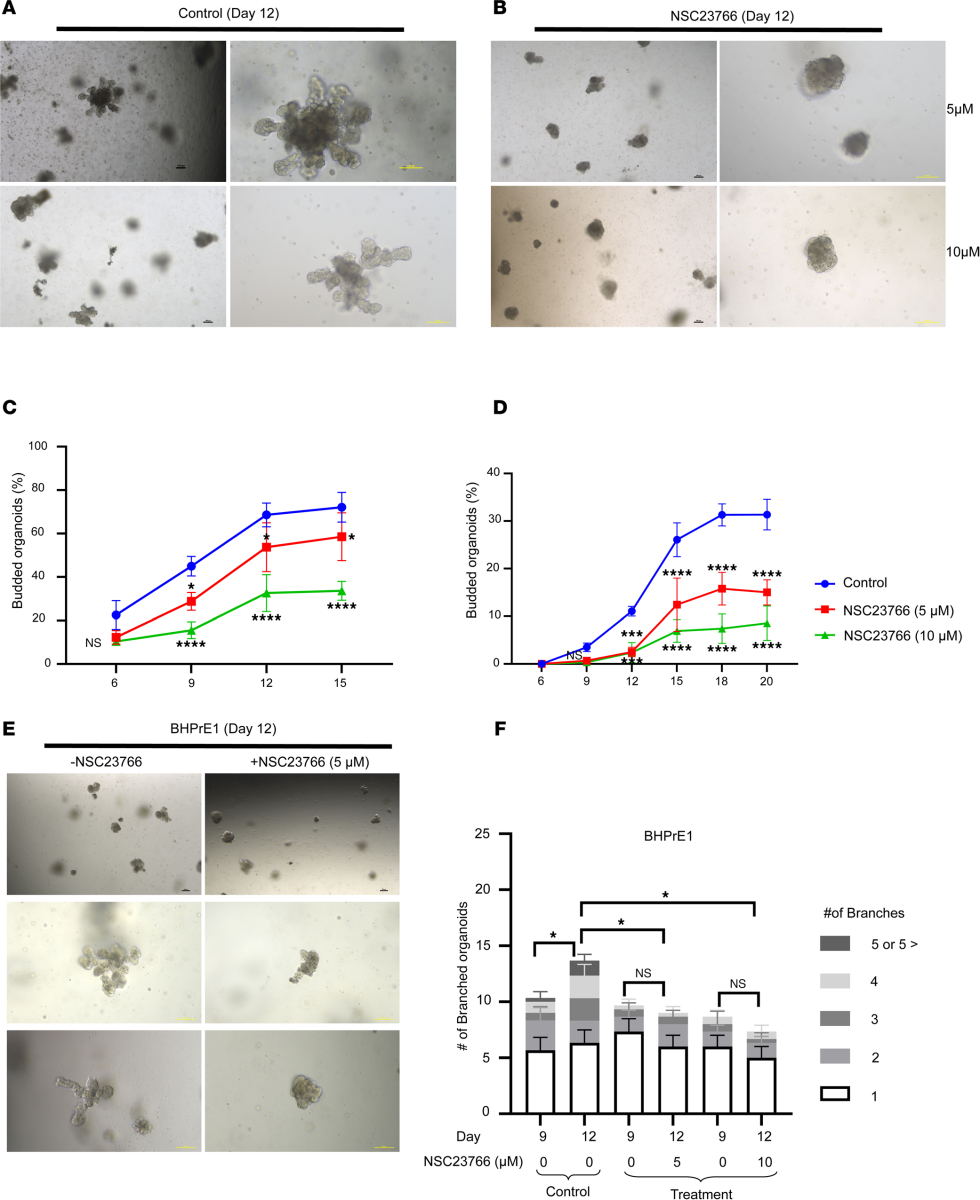
NSC23766 induced an impairment of prostatic organoid branching that phenocopied genetic knockdown of TIAM1. (**A**) Representative images taken on day 12 of BHPrE1 organoids. Scale bar: 100 μm. (**B**) Representative images taken on day 12 of BHPrE1 organoids with NSC23766 treatment starting on day 1 at 5 μM and 10 μM concentrations. Scale bar: 100 μm. (**C**) Quantification of the percentage of BHPrE1 budded organoids with NSC23766 treatment starting on day 1 at 5 μM and 10 μM concentrations compared with vehicle control. One-way ANOVA was used. (**D**) Quantification of the percentage of BHPrE1 branched organoids with NSC23766 treatment starting on day 1 at 5 μM and 10 μM concentrations compared with vehicle control. One-way ANOVA was used. (**E**) Representative images taken on day 12 of BHPrE1 organoids with NSC23766 treatment starting on day 9 at 5 μM concentration or vehicle control. Scale bar: 100 μm. (**F**) Quantification of the percentage of BHPrE1 branched organoids on day 9 and day 12, following treatment with NSC23766 on day 9. *n* = 3. Data are shown as mean ± SD. Two-way ANOVA was performed. *P < 0.05; ***P < 0.001; ****P < 0.0001. Each experiment was performed in 3 independent replicates.

NSC23766 has been shown to decrease branching phenotypes in other organ systems, including lung (24, 25) and mammary gland (26). To test the effect of NSC23766 on prostatic branching, organoid cultures of BHPrE1 cells were exposed to NSC23766. Following the exposure to NSC23766 from day 1, the cultures displayed a decrease in the number of budded and branched organoids in a dose-dependent manner (*P* < 0.05 and *P* < 0.0006; Figure 6, A–D). In another experiment, the effect of NSC23766 on BHPrE1 organoids with preexisting branches was evaluated. For this purpose, BHPrE1 organoid cultures were exposed to NSC23766 starting on day 9, a time point at which most of the organoids had begun forming branches, and effects on organoids were evaluated after 72 hours (day 12). While the number of branched organoids did not significantly increase in the treatment group, the vehicle control group displayed continued increase in the number of branched organoids from day 9 to day 12 (Figure 6, E and F). On day 12, the treatment group displayed no significant increase in the number of branched BHPrE1 organoids, whereas the control group showed a continued increase in the number of branched BHPrE1 organoids (*P* < 0.02; Figure 6, E and F). Similar effects on the branching of organoids were observed in RWPE-1 cells (Supplemental Figure 10) and in NHPrE1 cells (Supplemental Figure 11). Taken together, results demonstrate that NSC23766 exposure in a dose-dependent manner resulted in reduction of budding and branching in organoids of multiple human benign prostatic epithelial cells.

### NSC23766 exposure resulted in impaired organoid branching in stromal cocultures.

**Figure 7:**
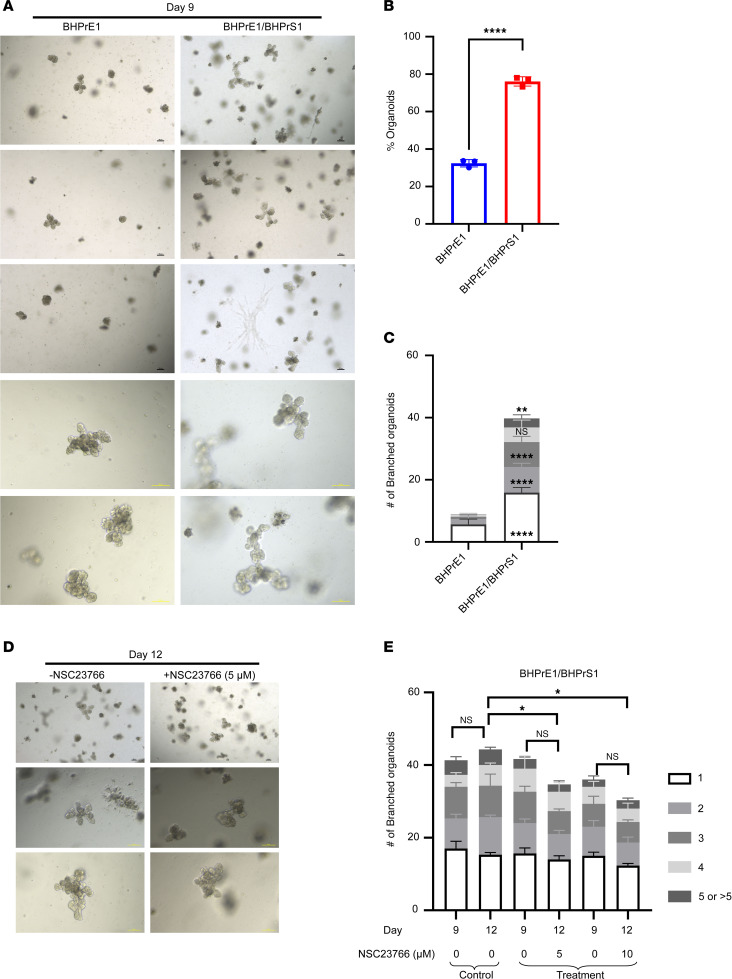
NSC23766 exposure resulted in impaired organoid branching in cocultures with the stroma. (**A**) Representative images taken on day 9 of BHPrE1 organoids in monoculture and coculture with stroma. Scale bar: 100 μm. (**B**) Quantification of the percentage of BHPrE1 organoids in monoculture and coculture with BHPrS1 cells. (**C**) Quantification of the number of branched organoids with multiple branches ranging from 1 to 5 branches in monoculture and coculture with stroma on day 9. n = 3. Data are shown as mean ± SD. Two-way ANOVA was performed. (**D**) Representative images taken on day 12 of BHPrE1 organoids in coculture with stroma treated with 5 μM NSC23766 or vehicle control. Scale bar: 100 μm. (**E**) Quantification of the number of BHPrE1 branched organoids in coculture with BHPrS1 cells on day 9 and day 12, following 5 μM NSC23766 treatment starting on day 9. *n* = 3 in each condition. Data are shown as mean ± SD. Two-way ANOVA was performed. *P < 0.05; **P < 0.01; ****P < 0.0001. Each experiment was performed in 2 independent replicates.

Since prostatic stroma has an established role in prostatic glandular differentiation and BPH pathophysiology (15, 27), and since coculture with stroma is reported to increase organoid branching, we evaluated the effects of NSC23766 on the branching of BHPrE1 organoids in coculture with BHPrS1 human prostatic stromal cells (Figure 7A and Supplemental Figure 12). Results demonstrate that coculture with stromal cells significantly increased the number of BHPrE1 organoids formed (*P* < 0.0001; Figure 7B). Furthermore, coculture significantly enhanced the branching of BHPrE1 organoids, including an increase in the number of organoids with multiple branches (Figure 7C). Next, the effects of NSC23766 on BHPrE1 organoids cocultured with stromal cells were evaluated. NSC23766 was added on day 9, and effects were assayed on day 12. On day 12, the treatment group displayed no significant increase in the number of branched BHPrE1 organoids, whereas the control group showed a continued increase in the number of branched BHPrE1 organoids (Figure 7, D and E). Furthermore, the total number of branched BHPrE1 organoids on day 12 in the treatment group was significantly reduced compared with controls even in stromal coculture conditions (*P* < 0.02; Figure 7, D and E). These results suggest NSC23766-mediated attenuating effects on prostatic budding and branching even in the physiological context of stromal cells.

### NSC23766 exposure resulted in growth inhibition of BPH patient-derived organoids.

**Figure 8:**
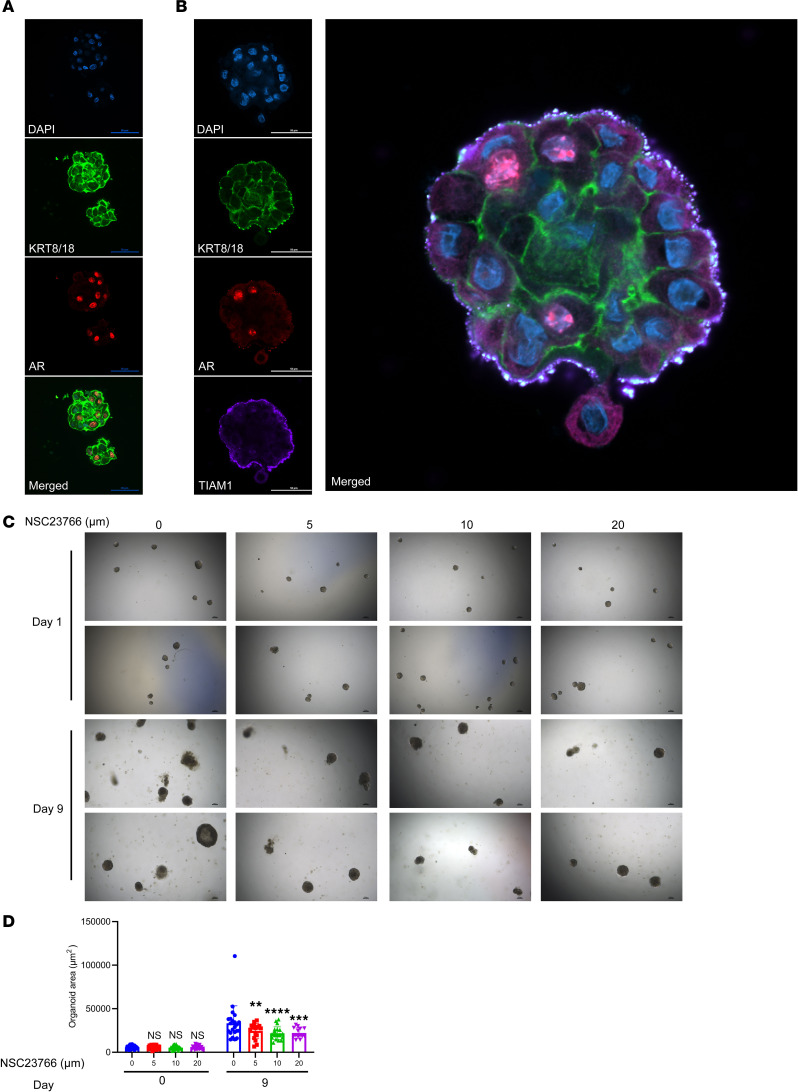
NSC23766 exposure resulted in growth inhibition of BPH patient-derived organoids (PDOs). (**A**) IF images of PDOs stained with DAPI, AR, and KRT8/18 (luminal marker). Scale bar: 50 μm. (**B**) IF images of PDOs stained with DAPI, AR, TIAM1, and KRT8/18. Scale bar: 50 μm. (**C**) Representative images of PDOs treated with 5, 10, and 20 μM NSC23766 compared with vehicle control at 4× original magnification. Scale bar: 100 μm. (**D**) Quantification of the PDOs showing the size of the PDOs on day zero (before treatment) and day 9 (after treatment). Paired 2-tailed Student’s *t* test was performed. **P *=* 0.0036; ***P *=* 0.0004; ****P < 0.0001.

Patient-derived organoids (PDOs) are 3D in vitro tissue culture models that may be useful for assessing the effects of pharmacological agents within perhaps a more physiological context than simple monolayer tissue culture in conditions for which no validated in vivo model systems are recognized. To further investigate pharmacological TIAM1/RAC1 signaling inhibition in the context of human BPH, we exposed BPH PDOs with varying concentrations of NSC23766 and assessed growth effects compared with vehicle controls. Both H&E staining (Supplemental Figure 13) and IF labeling of PDOs was performed for DAPI, AR, and KRT8/18 (Figure 8A) and DAPI, TIAM1, AR, and KRT8/18 (Figure 8B). Notably, exposure to NSC23766 (5, 10, 20 μM) caused inhibition of growth as assessed by organoid size compared with the vehicle control group (5 μM, *P* < 0.0036; 10 μM, *P* < 0.0001; 20 μM, *P =* 0.0004) (Figure 8, C and D, and Supplemental Figure 14). Furthermore, the size of PDOs showed a dose-dependent growth inhibition with increasing concentrations of NSC23766 (5 μM, *P* < 0.0036; 10 μM, *P* < 0.0001; 20 μM, *P =* 0.0004) (Figure 8D and Supplemental Figure 14). These results provide the first evidence to our knowledge of the potential of pharmacological small molecule inhibitors of Rac1 to inhibit prostate budding and branching growth in the context of BPH pathology.

## Discussion

In this study, multiple lines of evidence support that TIAM1 is a critical driver of prostatic budding and branching in BPH and suggest that targeting the TIAM1/RAC1 axis may be a potentially effective treatment strategy. (a) TIAM1 was found to be elevated in a transcriptomic signature derived from bioinformatic analysis of 3 independent BPH patient cohort datasets, with elevated TIAM1 protein levels confirmed in a fourth. (b) Both IPA and CMap analyses of the BPH transcriptomic signature indicated TIAM1/RAC1 axis signaling as a potential lead pathway for therapeutic targeting. (c) *TIAM1* knockdown and overexpression studies revealed that the branching phenotype of prostatic organoids is dependent on TIAM1 expression. (d) In both epithelial cell organoids and epithelial cells/stromal coculture conditions, pharmacological RAC1 inhibition using NSC23766 attenuated branching of human prostatic organoids. (e) Last, growth inhibition was observed in patient-derived BPH organoids treated with NSC23766. Overall, identification of TIAM1OE in human BPH cohorts via bioinformatic and protein analyses was followed by complementary studies of genetic TIAM1 knockdown/overexpression and pharmacological-based RAC1 inhibition in experimental in vitro BPH models to identify a new role for the TIAM1/RAC1 axis in human BPH.

While the experimental modeling data reported here is consistent with a role for TIAM1 and RAC1 in the development of clinical BPH, it is recognized that the bioinformatic analysis of clinical BPH data has several inherent limitations. These include that the datasets relied on BPH samples from 1) patients of varying past medical treatment histories, 2) had variances in the control tissue used (TZ versus PZ), and 3) had variances in postharvest tissue processing methodologies (RNA isolation from paraffin blocks versus frozen tissue). Despite these variances, integration of the datasets permitted the analysis of a greater number of more broadly sampled BPH tissue specimens than would otherwise have been possible to serve as the initiating basis for the current study.

In the ontogeny of humans and other organisms, budding and branching morphogenesis underlines the development of numerous organs, including the prostate. Although growth and arborization is typically repressed in the adult prostate, molecular cues are believed to reactivate this developmental program in BPH, leading to its hyperplastic phenotype (14, 28). However, the molecular triggers leading to the reactivation of branching morphogenesis in BPH remain elusive.

Whereas previous studies that focused on prostate ontogeny have utilized rat and mouse models (29–32), recent reports on prostate pathology have utilized organoid models (15, 16). Organoids, which are miniature and simplified versions of organs, serve as valuable tools to study the biology of a signaling pathway in a physiological context. Therefore, we investigated the effects of TIAM1 inhibition on prostatic branching phenotype utilizing organoids formed from human benign prostatic epithelial cell lines. Such epithelial cells organize into basal and luminal layers, express androgen receptor (AR) and AR target genes, and eventually bud and branch to form prostatic glands in 3D culture (15).

Our results demonstrate that genetic knockdown of *TIAM1* had a marked suppressive effect on the prostatic branching phenotype, suggesting that TIAM1 may be critical for the reactivated prostatic branching that results in the hyperplastic BPH phenotype. Furthermore, the study of organoid cultures of human prostatic cell lines — either through genetic knockdown or use of Rac1 inhibitor, NSC23766 — allowed us to begin initial investigations on the biology of the TIAM1/RAC1 signaling pathway in BPH, with the results from these models corroborated in PDOs.

Branching morphogenesis is a complex process that often involves crosstalk among multiple signaling pathways (33–35). For example, interaction between Rac1 and RhoA in regulating cell shape drives the invagination morphogenesis of epithelial cells in the developing mouse lens (36).

Notably, a study on rat prostate development highlights a critical role of MEK/ERK and RHO signaling pathways in prostatic growth and branching. These findings underscore the need for future investigations to explore how targeting multiple nodes within these signaling networks could offer a more effective therapeutic strategy for diseases involving abnormal branching morphogenesis, including BPH.

The role of stroma in prostatic pathology is well established. It has been reported previously that NSC23766 leads to a decrease in proliferation of prostatic stromal cells (37). It is hypothesized that the stroma is a crucial player in BPH budding and branching since paracrine interactions between stroma and epithelia play an established role during embryonic development of the prostate (15). However, the molecular mechanisms that drive these stromal-epithelial interactions in BPH pathogenesis remain elusive. The role of the stroma in the pathophysiology of the BPH has now been recognized, and the effect of the stroma on therapeutic responses has also been well documented (15, 27). In accordance with these reports, our study shows that NSC23766-mediated impairment of the prostatic budding and branching phenotypes is observed even in the presence of stroma.

TIAM1 is a RAC1-specific GEF (38). The majority of the cellular activity of TIAM1 is attributed to its activation of the small GTPase, Rac1, switching it from an inactive GDP-bound state to an active GTP-bound state. Consistent with these observations, our results show that both *TIAM1* knockdown and pharmacological inhibition of TIAM1/RAC1 using NSC23766 led to decreased levels of active RAC1, thereby providing validation of TIAM1/RAC1 signaling in prostatic biology.

TIAM1-dependent activation of RAC1 plays fundamental roles in a variety of cellular processes, including cellular proliferation, migration, differentiation (39) and inflammation (22). In epithelial cells, TIAM1 is reported to facilitate formation of lamellipodia and filopodia (40). In keratinocytes, TIAM1 controls polarization of migratory cells (41). Knockdown and overexpression studies have shown that *TIAM1* regulates cell proliferation, survival and migration. Of relevance to our study, TIAM1 is reported to mediate neurite outgrowth, branching and angiogenesis (22).

Of note, NSC23766 has been reported to impair branching morphogenesis in other organ systems, and treatment with NSC23766 resulted in decreased lung branching in mouse and human explants (24, 25). The current study is the first to our knowledge to report the effects of NSC23766-mediated TIAM1-RAC1 inhibition on BPH budding and branching. Previous studies on NSC23766 in the context of other pathologies have resulted in cellular toxicities at concentrations approximately 10-fold higher (20) than those we found to be effective in reducing budding and branching in prostatic organoids. Furthermore, prior animal studies have demonstrated that NSC23766 can be used in vivo without apparent evidence of organ toxicity in mice (36, 42, 43). Future studies will be needed to determine the clinical applicability of RAC1 and/or TIAM1 inhibitors for BPH treatment.

In summary, our study employed both bioinformatic analyses on clinical BPH samples and laboratory modeling using human prostatic cell lines and BPH PDOs to identify and demonstrate (a) TIAM1 as a molecular driver of the branching phenotype in BPH and (b) inhibition of the TIAM1/RAC1 axis as a possible therapeutic approach to the effective treatment of BPH.

## Methods

### Sex as a biological variable.

Our study exclusively involved males because the prostate is a male-specific organ and disorders of the prostate are only relevant in males.

### RNA-Seq data processing and analysis.

Three mRNA expression datasets (44–46) were obtained from the Genotypes and Phenotypes (dbGap) (https://www.ncbi.nlm.nih.gov/gap/) and the Gene Expression Omnibus (GEO) databases (https://www.ncbi.nlm.nih.gov/geo/*)*.

Supplemental Table 1 outlines the information regarding the datasets utilized in the current study. Briefly, Jin et al. (46) used 30 BPH and 14 control samples for the RNA-Seq. The TZ from BPH was collected at the time of bladder outlet obstruction surgery for the treatment of LUTS. The control tissue used was benign TZ tissue from patients that underwent radical prostatectomy for low-volume, low-grade prostate cancer that was localized to the PZ. Liu et al. (44) utilized 18 BPH and 4 control samples for the RNA-Seq (accession no. GSE132714). Prostate tissue from TZ of patients with BPH was utilized for the study. The controls for RNA-Seq were obtained from men undergoing radical prostatectomy for prostate cancer without BPH. Middleton et al. (45) utilized 37 (28 were available in dbGap) BPH and 19 (18 were available in dbGap) controls, acquired concurrent BPH tissues from radical prostatectomies for prostate cancer; control tissue was obtained from normal PZ regions of prostates without other zonal pathologic changes (accession no. phs001698.v1.p1).

The DNASTAR SeqMan NGen assembler (DNASTAR 2016) was used to map the FASTQ files to the human reference genome (GRCh38). The assembled RNA-Seq reads were normalized by the reads per kilobase per million mapped reads (RPKM) method. Differences in gene expression levels were analyzed using Fisher’s exact test in the ArrayStar software package (DNASTAR 2016). DEGs were filtered with a fold-change of ≥ |1.5| and FDR of < 0.05, using Bonferroni-Hochberg correction method. An online Venn analysis tool (http://bioinformatics.psb.ugent.be/webtools/Venn/) was utilized to overlap the 3 sets of DEGs in order to arrive at the cDEGs — i.e., the shared BPH transcriptomic signature. The heatmaps and box plots were generated using Pheatmap (https://cran.r-project.org/web/packages/pheatmap/index.html) and ggPlot2 (https://cran.r-project.org/web/packages/ggplot2/index.html). DAVID (https://david.ncifcrf.gov/) (47) was used to analyze GO to generate functional annotations for cellular and molecular functions, including CC, BP, and molecular functions. Reactome analysis was used to determine the biological functions of the cDEGs, and putative upstream regulators were determined by IPA (QIAGEN Inc., https://digitalinsights.qiagen.com/IPA) (48). cDEGs were interrogated for potential therapeutic candidate compounds using CMap (L1000 platform; https://clue.io/) (18), a widely used in silico drug-screening tool that uses transcriptional expression data, both upregulated and downregulated genes, to investigate relationships between disease conditions and therapeutic modalities on the basis of altered BP or pathways.

### Cell culture.

Human benign prostatic cell lines (Supplemental Table 2), BPH-1, BHPrE1, BHPrS1, and NHPrE1, were provided by Simon Hayward, North Shore University Health System Research Institute. Normal human prostate epithelial cell line, RWPE-1, was purchased from American Type Culture Collection (catalog CRL-3607). Cell lines were maintained at 37°C in a humidified CO_2_ (5%) incubator. BPH-1 cells were maintained in Roswell Park Memorial Institute Medium (RPMI; Corning, catalog 10040CV) supplemented with 10% Cosmic Calf Serum (CCS; HyClone, catalog 16777-244) and 1% penicillin and streptomycin. BHPrE1 cells were maintained in DMEM F-12 (Corning, catalog 10090CV), 5% FBS (Thermo Fisher Scientific, catalog 10437-028), 1% penicillin and streptomycin (Cytiva, catalog SV30010), 0.4% Bovine Pituitary Extract (BPE; Thermo Fisher Scientific, catalog 13028014), 10 ng/mL Epidermal Growth Factor, (EGF; MilliporeSigma, catalog E4127) and 1% Insulin-Transferrin-Selenium (ITS; Thermo Fisher Scientific, catalog 41400045); BHPrS1 cells were maintained in RPMI-1640 with 5% FBS and 1% penicillin and streptomycin; RWPE-1 cells were maintained in keratinocyte medium (Thermo Fisher Scientific, catalog 10724011) containing BPE and EGF. The cell lines used for this study were intermittently evaluated in-house for mycoplasma contamination. For transient overexpression of TIAM1 protein, BHPrE1 cells were transfected with a full-length pcDNA-Myc-tagged TIAM1 (a gift from Kevin Pruitt, TTUHSC) (49) or the empty vector, using Lipofectamine 2000 transfection reagent (Thermo Fisher Scientific, catalog 11668027). For stable overexpression of TIAM1 protein, BHPrE1 cells were transduced with pMXs3-TIAM1 (a gift from David Sabatini; Addgene plasmid no. 86143; http://n2t.net/addgene:86143; RRID:Addgene_86143) (50) or empty vector. NSC23766 is a small molecule that blocks the activation of RAC1 protein by inhibiting TIAM1 activity (28, 29). NSC23766 was purchased from Tocris (catalog 21-611-0R), reconstituted in molecular grade water to 100 mM, aliquoted, and stored at –80°C.

### RNA isolation, cDNA synthesis, and qPCR.

Total RNAs were extracted from human benign prostatic cells using the RNeasy Mini Kit as per manufacturer’s protocol (QIAGEN, catalog 74104). cDNA was synthesized using a high-capacity RNA to cDNA kit (Applied Biosystems, catalog 4368814) as per the manufacturer’s protocol. qPCR was performed using PowerUp SYBR Green Master Mix (Invitrogen, catalog A25742) with specific gene primers synthesized by Integrated DNA Technologies (*hTIAM1* forward [F]: 5′AAGACGTACTCAGGCCATGTCC3′, *hTIAM1* reverse [R]: 5′GACCCAAATGTCGCAGCAG3′, *hACTIN* F: 5′CTGGAACGGTGAAGGTGACA3′, *hACTIN* R: 5′AAGGGACTTCCTGTAACAATGCA3′). The qPCR analysis was performed on the QuantStudio 12K Flex platform.

### Cell lysate preparation and Western blot analysis.

Total protein lysates were prepared from the cells followed by protein quantification using the DC Protein Assay Kit (Bio-Rad, catalog 5000001) as described previously. Extracted proteins were resolved on a 10% SDS-PAGE gel and electroblotted onto a PVDF membrane (Sigma-Aldrich, catalog IPVH00005). The membranes were blocked with 1× Blocker FL Fluorescent Blocking Buffer (Thermo Fisher Scientific, catalog 37565) followed by incubation with the respective primary antibodies overnight at 4°C. A detailed list of the antibodies used in this study is provided in the supplementary information (Supplemental Table 3). Subsequent to washing with TBST (0.1% Tween), the membranes were probed with HRP-conjugated secondary antibodies at 1:2,000 dilution (rabbit/mouse, Cell Signaling Technology) for 1 hour at room temperature (RT). Bands were visualized using the SuperSignal West Pico PLUS Chemiluminescent kit (Thermo Fisher Scientific, catalog 34580) under ChemiDoc Touch Imaging System (Bio-Rad). β-Actin was used as the loading control.

### IHC of human prostate tissue samples.

After obtaining IRB approval, IHC analysis was performed on deidentified, paraffin-embedded human prostate tissues: BPH (*n* = 14) and control tissues (*n* = 10). To reduce batch effects, all samples were processed together for IHC analysis. In brief, slides were incubated in xylene (Thermo Fisher Scientific, catalog X3S-4) 3 times (5 minutes each) for deparaffinization. Following a series of 5-minute incubations in ethanol at decreasing concentrations (100%–10%) and 3 phosphate-buffered saline (PBS) washes for 5 minutes each, slide sections were hydrated by rinsing under running water. Using 1× antigen unmasking buffer (Vector Laboratories, catalog H-3300), antigen unmasking was carried out in a decloaking chamber (Oster). This was followed by blocking of endogenous peroxidases using Bloxall (Vector Laboratories, catalog SP-6000). Next, Avidin and Biotin blocking (Vector Laboratories, catalog SP-2001) was performed followed by a PBS wash. Goat serum (Vector Laboratories, catalog S-1000) at a dilution of 5% in PBS was used to block the tissue section. Next, the slides were incubated overnight with anti-TIAM1 primary at 4°C in a humidified chamber. The details regarding the antibodies used in this study are provided in the supplementary information (Supplemental Table 3). The following day, 3 PBS washes were performed for 5 minutes each, and the slides were incubated for 1 hour with the secondary antibody (Vector Laboratories, BA-1000, 1:200 dilution), followed by 3 PBS washes for 5 minutes each.

The slides were then incubated with premixed avidin biotin complex (Vector Lab, catalog PK-6101) for 45 minutes and washed twice with PBS. For the development of color, peroxidase substrate (Vector Laboratories, catalog SK-4105) was added, followed by washing the slides with water, counterstaining using hematoxylin (Vector Laboratories, catalog H-3401), and then rinsing again with water before mounting with Vectamount (Vector Laboratories, catalog H-5000). The specificity of the TIAM1 antibody was validated by experiments where genetic knockdown of TIAM1 resulted in loss of TIAM1 protein expression as visualized by IF staining (Supplemental Figure 3).

### Image acquisition and analysis.

The IHC stained slides were analyzed and scored based on the immunoreactive score (IRS) (51). In short, IRS is used to quantify the IHC staining results by evaluating the percentage of positive cells and the intensity of staining. For this analysis, the pathologist visually estimated the proportion of cells showing immunoreactivity (positive cells) and assessed the strength of the staining intensity. The IHC score was calculated as IRS = (percentage of positive cells) × (intensity of staining). The images were acquired using Olympus XI83 microscope.

### Proliferation assay.

Cell proliferation was measured using WST-1 (water-soluble tetrazolium) assay (Sigma-Aldrich, catalog 501003295) according to standard manufacturer’s protocol. Cells (2,000/well) were seeded in a 96-well plate with 100 μL of culture medium and incubated at 5% CO_2_, 37°C overnight. The following day, NSC23766 (1.25–200 μM) or vehicle control (molecular grade water) were administered, and plates were reincubated for 5 days. Cell proliferation was examined at 24, 48, 72, 96, and 120 hours using WST-1 reagent. To assess cell proliferation, absorbance of the samples was measured at 450 nm using an iMark microplate reader (Bio-Rad) and absorbance normalized by comparison with controls and by subtracting the absorbance of a background blank per the manufacturer’s protocol.

### Apoptosis assay.

Apoptosis was evaluated using the annexin V–FITC Apoptosis Detection Kit (Invitrogen, 88-8005-74). Cells were seeded at 50,000 cells per well in 6-well plates (Corning, 3516) in 2 mL target media. Cells were allowed to incubate overnight, and then NSC23766 was added at a 10 μM final concentration; controls received an equivalent volume of water. Cells were reincubated for 48 hours (BHPrE1, NHPrE1, and BHPrS1). Cells were harvested by trypsinization, washed twice with cold PBS, and resuspended in 1× annexin V binding buffer. For staining, 5 μL of annexin V–FITC (Invitrogen, catalog 11-8005-74) and 1 μL of PI (Invitrogen, catalog 00-6990-42) were added to 100 μL of cell suspension. Cells were incubated for 15 minutes at RT in the dark, according to the manufacturer’s instructions. Flow cytometric analysis was performed using a Beckman flow cytometer; 10,000 events were collected per sample. The gating strategy was designed to distinguish live cells, early apoptotic cells, and necrotic cells based on annexin V–FITC and PI staining. Data analysis was performed using Kaluza software to quantify the percentage of live, early apoptotic, and late apoptotic/necrotic cells.

### Organoid assay.

For the monoculture organoid assay, 700 cells/well were mixed in 300 μL of 5 μg/mL of Corning Matrigel GFR Basement Membrane Matrix (Thermo Fisher Scientific; catalog CB40230) in the organoid media containing KSFM medium and 10 nM DHT and 5% charcoal stripped serum (Thermo Fisher Scientific, catalog A5669501) and seeded in 24 well plates (Costar, 3524). The assay was performed as per the manufacturer’s protocol (Corning Life Sciences) and previously published protocols (16, 52, 53). The cultures were maintained in 500 μL of organoid media that was changed every 2–3 days. For coculture experiments, 700 epithelial cells were seeded in Matrigel along with 1,400 stromal cells (BHPrS1). The cocultures were maintained in 500 μL medium changed every 2–3 days. Assessment of branching was after the method of McCray et al. (53). The organoids were analyzed for size, number, buds, and branches using the inbuilt EIS microscope software (Nikon microscope) and ImageJ software (NIH).

### PDO assay.

Under an IRB-approved protocol, organoid cultures were performed on deidentified human prostate tissues from the TZ of patients who underwent prostatectomy, PDO1 (63-year-old patient diagnosed with BPH), PDO2 (74-year-old patient diagnosed with BPH), PDO3 (63-year-old patient diagnosed with BPH), and PDO4 (77-year-old patient diagnosed with BPH). Tissues were assessed by the pathologist to be free of cancer. Tissue digestion was performed as, briefly, tissue (~200 mg) was placed in a petri dish with 2–4 mL of digestion solution containing 1 mg/mL Collagenase I (Thermo Fisher Scientific, catalog 17100-017) and 0.2 U/mL dispase-II (MilliporeSigma, catalog SCM133) dissolved in 50 mL serum-free antibiotic-free DMEM F12 medium. The tissue was minced using a scalpel and incubated with the digestion solution, at 37°C for 3–4 hours on a shaker at 200 rpm. Then, the sample was centrifuged at 300*g* at RT, and the medium was aspirated. The sample was then incubated with 5–10 mL of TrypLE (Thermo Fisher Scientific, catalog 12604-013) at 37°C for 5–10 minutes on a shaker at 200 rpm. After neutralizing TrypLE with 10 mL RPMI, the suspension was passed slowly through a 20 G needle, followed by 70 μm and 40 μm cell strainers. The sample was centrifuged again at 300*g*, and the supernatant was removed. The sample pellet was resuspended in 5–10 mL of RBC lysis buffer (Thermo Fisher Scientific, catalog 00-4300-54) and left at RT for 5 minutes. After RBC neutralization with PBS, the cells were resuspended in 200 μL of organoid medium, and 700 cells per well were seeded in a 24-well plate for organoid culture as described in the organoid assay section. After 7–10 days of culture, the PDOs were treated with 10 μM NSC23766 or vehicle control for another 9 days following media and treatment change every 2 days.

### PDO harvesting, sectioning, and staining.

After 9 days of treatment, PDOs were collected into a 15 mL centrifuge tube. The organoids were then washed with 1 mL cold PBS to remove the residual medium. The PDOs were then centrifuged at 280*g* for 5 minutes at 4°C, and the supernatant was discarded. The pellet was resuspended in 5 mL cold DMEM media containing 20 mM HEPES (HyClone, catalog SH30237.01) before being gently pipetted up and down to mix the remaining Matrigel with medium. The suspension was then centrifuged at 200*g* for 5 minutes at 4°C. The collected organoids were mixed with 200 μL Matrigel and frozen at –80°C for 10 minutes before being covered with tissue freezing media (FBS with 10% DMSO) (General Data, catalog TFM-5) to preserve their structural integrity for analysis. Embedded PDOs were sectioned at the TTUHSC Molecular Biology Core Facility at TTUHSC into 5 μm–thick slices using a cryostat.

### Lentiviral vector.

HEK293T cells (1.2 × 10^7^) were cotransfected with either lentiviral vector containing constructs, pLKO.1-Neo-CMV-tGFP *TIAM1* shRNA plasmid (Sigma-Aldrich, catalog 07202334MN, TRCN0000256946 that targets “TTCGAAGGCTGTACGTGAATA”) or pLKO.1-Neo-CMV-tGFP-Scramble shRNA plasmid (Addgene no. 136035 as scramble control). Lentiviral vectors were packaged using packaging plasmid (pCMV ∆R8.2 dvpr, Addgene no. 8455) and the envelope plasmid (pCMV-VSV-G, Addgene no. 8454). After 72 hours of transfection, the virus-containing media were collected and filtered and used for the BHPrE1 cells. After 3 days, cells were treated with 500 μg/mL neomycin to obtain stable *TIAM1-*knockdown clones.

### Active RAC1 detection assay.

The activation of RAC1 was determined using active RAC1 detection kit (catalog 8853, Cell Signaling Technology) according to the manufacturer’s protocol. In brief, cultured cells were washed with ice-cold 1× PBS and lysed with 1× lysis buffer containing 1 mM phenylmethanesulfonyl fluoride (catalog 8815, Cell Signaling Technology) followed by centrifugation at 16,000*g* for 15 minutes at 4°C. The supernatant was then incubated with glutathione resin and GST-PAK1-PBD (a fusion protein) supplied with the kit for 1 hour at 4°C with constant rotation. After washing the resin with 1× cell lysis/binding/wash buffer, the bound GTP-RAC1 was eluted using reducing sample buffer. The amount of bound GTP-RAC1 was analyzed by Western blot using RAC1 mouse mAb (catalog 8631, 1:1,000, Cell Signaling Technology). The cell lysate was used to detect total RAC1 (catalog 05-389, MilliporeSigma), and β-actin (catalog sc-47778, Santa Cruz Biotechnology Inc.) was used as the loading control.

### Statistics.

Statistical analyses were performed using GraphPad Prism version 9.0.1 (GraphPad Software). The unpaired 2-tailed Student’s *t* test was employed for comparing 2 groups, and 1-way ANOVA and 2-way ANOVA were utilized to determine statistical significance for 3 or more groups followed by Tukey’s post hoc test. Differences were considered statistically significant when *P* < 0.05. All the experiments were done, *n* = 3 and data are presented as mean ± SD.

### Study approval.

The study was approved by the IRB of TTUHSC.

### Data availability.

We used raw data from publicly available datasets deposited to the GEO Database (www.ncbi.nlm.nih.gov/geo/) and the dbGap (https://www.ncbi.nlm.nih.gov/gap/). Quantitative data supporting the manuscript figures are included in the Supporting Data Values file.

## Author contributions

HK designed and conducted the majority of experiments, methodology development, data analysis, and manuscript preparation. SD, MD, MR, GKP, DL, MKJ, and LB contributed to data acquisition. MF, IW, AH, WDR, and RJM provided resources. RJM and BJM contributed to data interpretation and manuscript review and editing. SN and MT were responsible for conceptualization, resource provision, project supervision, data interpretation, funding acquisition, and manuscript preparation and editing. All authors read and approved the final manuscript.

## Supplementary Material

Supplemental data

Unedited blot and gel images

Supplemental table 4

Supporting data values

## Figures and Tables

**Table 1:**
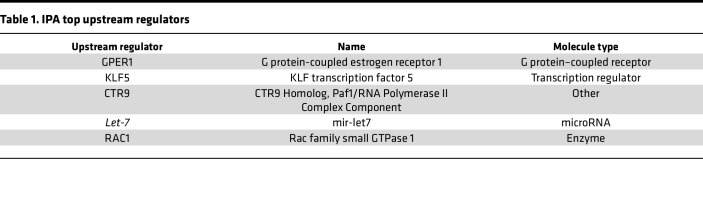
IPA top upstream regulators

## Version 1 05/20/2025

In-Press Preview

## Version 2 06/23/2025

Electronic publication
