# Supplementary material for: TIAM1 drives prostatic branching phenotype and is a potential therapeutic target for benign prostatic hyperplasia
Source: JCI Insight. 2025 May 20;10(12):e188062. doi: 10.1172/jci.insight.188062 (PMC12220968; doi:10.1172/jci.insight.188062)
Supplement: Supplemental data [file jciinsight-10-188062-s141.pdf]

**Supplementary Figures:**

**Table S1: RNA-seq datasets utilized in the study for identifying the differentially expressed genes in BPH.**

| Study Group | Sample Size | Specimens | Treatment |
|---|---|---|---|
| Middleton et al (45) | BPH: 37 (28)<br>Control: 19 (18) | Formalin-fixed, paraffin-embedded blocks of radical prostatectomies of prostate cancer that had concurrent BPH (BPH in transition zone, and normal prostate from peripheral zone as control). | Patients were not on 5-α-reductase inhibitors or α-blockers. |
| Liu et al (44) | BPH: 18<br>Control: 4 | Frozen tissue from the transition zone of BPH patient. Controls from the transition zones of men undergoing radical prostatectomy for prostate cancer without BPH. | Patients were exposed to 5-alpha reductase inhibitors and α-blockers. |
| Jin et al (46) | BPH: 30<br>Control:14 | Frozen benign tissue from transition zone of patients who failed medical therapy and underwent surgical treatment for BPH, referred as Surgical BPH. Control, referred as Incidental BPH, is from the transition zone of men undergoing radical prostatectomy for low-volume, low grade prostate cancer. | Patients were exposed to 5-alpha reductase inhibitors and α-blockers. |

**Table S2: The list of cell lines used in the study.**

| | |
|---|---|
| BPH-1 | Immortalized but not transformed Human prostate epithelial cell line |
| BHPrE1 | Spontaneously immortalized adult non-tumorigenic human prostate epithelial cell line |
| NHPrE1 | Spontaneously immortalized adult non-tumorigenic human prostate epithelial cell line |
| RWPE-1 | Immortalized benign human prostate epithelial cell line |
| BHPrS1 | Immortalized human BPH stromal cell line |
| WPMY | Immortalized benign human prostate stromal cell line |

**Table S3: The list of the antibodies used in the study.**

| Antibody | Recourse | Dilution used |
|---|---|---|
| KRT5 | Cell Signaling, 71536S | 1:500 |
| KRT8/18 | Cell Signaling, 4546S | 1:500 |
| KRT14 | Santa Cruz Biotechnology, LL001 | 1:250 |
| TIAM1 | Abcam, ab211518 | 1:500 (IF), 1:1000 (WB) |
| TIAM1 | Fisher Scientific, PA5-142082 | 1:500 |
| Alexa Fluor 568 goat anti-mouse | Invitrogen, A11004 | 1:1000 |
| Alexa Fluor 568 goat anti-rabbit | Invitrogen, A11011 | 1:1000 |
| Alexa Fluor 647 goat anti-mouse | Invitrogen, 32728TR | 1:1000 |
| Alexa Fluor 647 Monkey anti-goat | Invitrogen, A21447 | 1:1000 |
| Alexa Fluor 488 goat anti-mouse | Invitrogen, A11001 | 1:1000 |
| B-ACTIN | Santa Cruz Biotechnology, sc-47778 | 1:2500 |
| AR | Cell Signaling, 5153S | 1:500 |

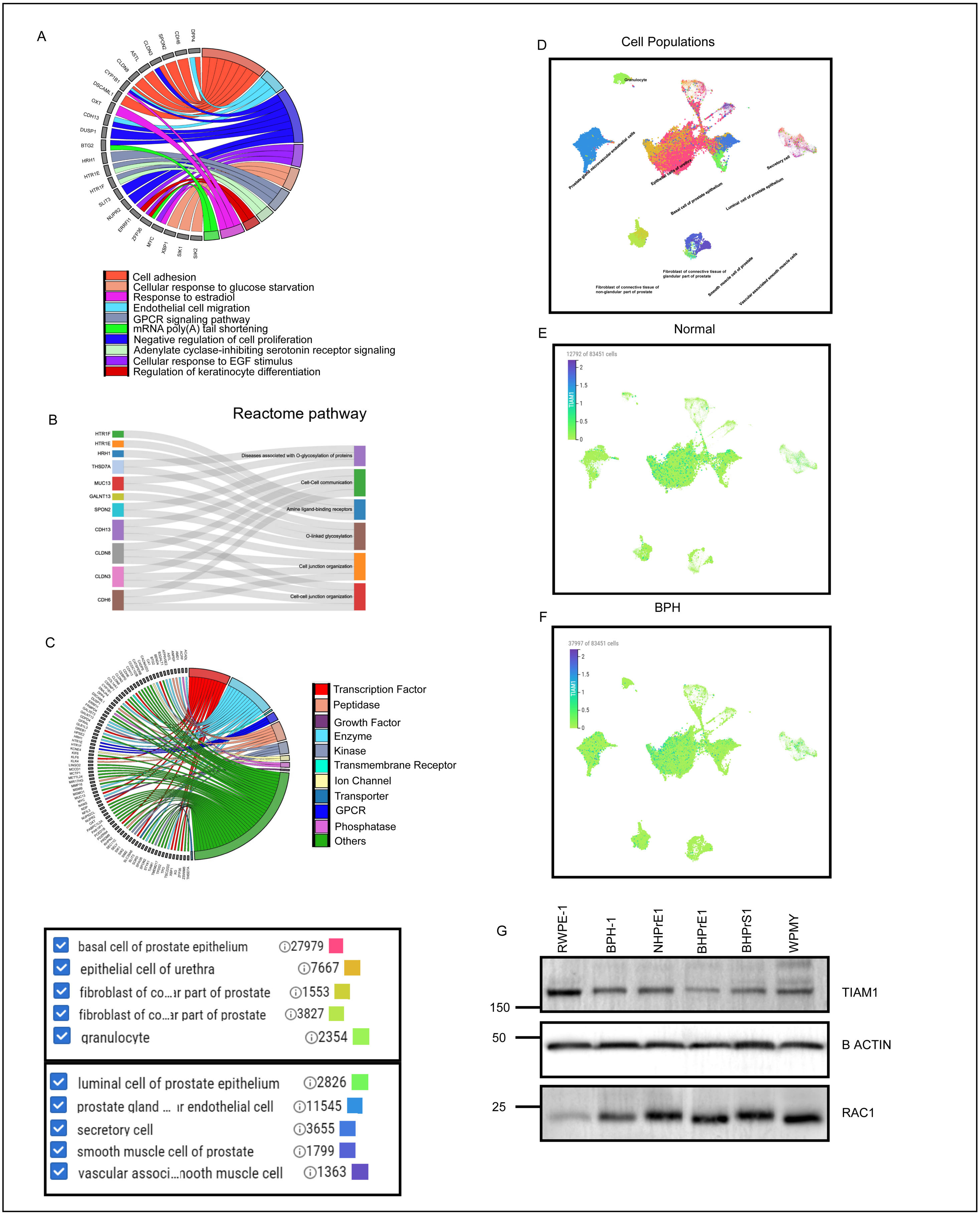
A
DPP4
CDH6
SPON2
CLDN3
ASTL
CLDN8
CYP1B1
DSCAML1
OXT
CDH13
DUSP1
BTG2
HRH1
HTR1E
HTR1F
SLIT3
NUPR2
ERRFI1
ZFP36
MYC
XBP1
SIK1
SIK2
Cell adhesion
Cellular response to glucose starvation
Response to estradiol
Endothelial cell migration
GPCR signaling pathway
mRNA poly(A) tail shortening
Negative regulation of cell proliferation
Adenylate cyclase-inhibiting serotonin receptor signaling
Cellular response to EGF stimulus
Regulation of keratinocyte differentiation
B
Reactome pathway
HTR1F
HTR1E
HRH1
THSD7A
MUC13
GALNT13
SPON2
CDH13
CLDN8
CLDN3
CDH6
Diseases associated with O-glycosylation of proteins
Cell-Cell communication
Amine ligand-binding receptors
O-linked glycosylation
Cell junction organization
Cell-cell junction organization
C
Transcription Factor
Peptidase
Growth Factor
Enzyme
Kinase
Transmembrane Receptor
Ion Channel
Transporter
GPCR
Phosphatase
Others
basal cell of prostate epithelium 27979
epithelial cell of urethra 7667
fibroblast of co...ar part of prostate 1553
fibroblast of co...ar part of prostate 3827
granulocyte 2354
luminal cell of prostate epithelium 2826
prostate gland ... ar endothelial cell 11545
secretory cell 3655
smooth muscle cell of prostate 1799
vascular associ...nooth muscle cell 1363
D
Cell Populations
Granulocyte
Prostate gland microvascular endothelial cells
Epithelial cells of urethra
Basal cell of prostate epithelium
Secretory cell
Luminal cell of prostate epithelium
Fibroblast of connective tissue of glandular part of prostate
Fibroblast of connective tissue of non-glandular part of prostate
Smooth muscle cell of prostate
Vascular associated smooth muscle cells
E
Normal
12792 of 83451 cells
TIAM1
2
1.5
1
0.5
0
F
BPH
37997 of 83451 cells
TIAM1
2
1.5
1
0.5
0
G
RWPE-1
BPH-1
NHPrE1
BHPrE1
BHPrS1
WPMY
TIAM1
150
50
B ACTIN
25
RAC1

**Figure S1: Additional data on the BPH transcriptomic signature and TIAM1 expression. A)** Circos plot illustrating the correlation between biological processes and their associated cDEGs (*P* < 0.05). **B)** Reactome pathway analysis showing the significantly enriched Reactome pathways from the 84 cDEGs. **C)** Circos plot illustrating the correlation between cDEGs and their functional categories, including transcription factors, peptidases, growth factors, enzymes, kinases, transmembrane receptors, ion channels, transporters, G-protein coupled receptors, and phosphatases. **D)** Overview of the key prostate cell populations analyzed for TIAM1 expression. **E)** TIAM1 expression in different cell populations of Normal samples. **F)** TIAM1 expression across different cell populations in BPH samples. **G)** Expression of TIAM1 and RAC1 in different benign prostatic cell lines.

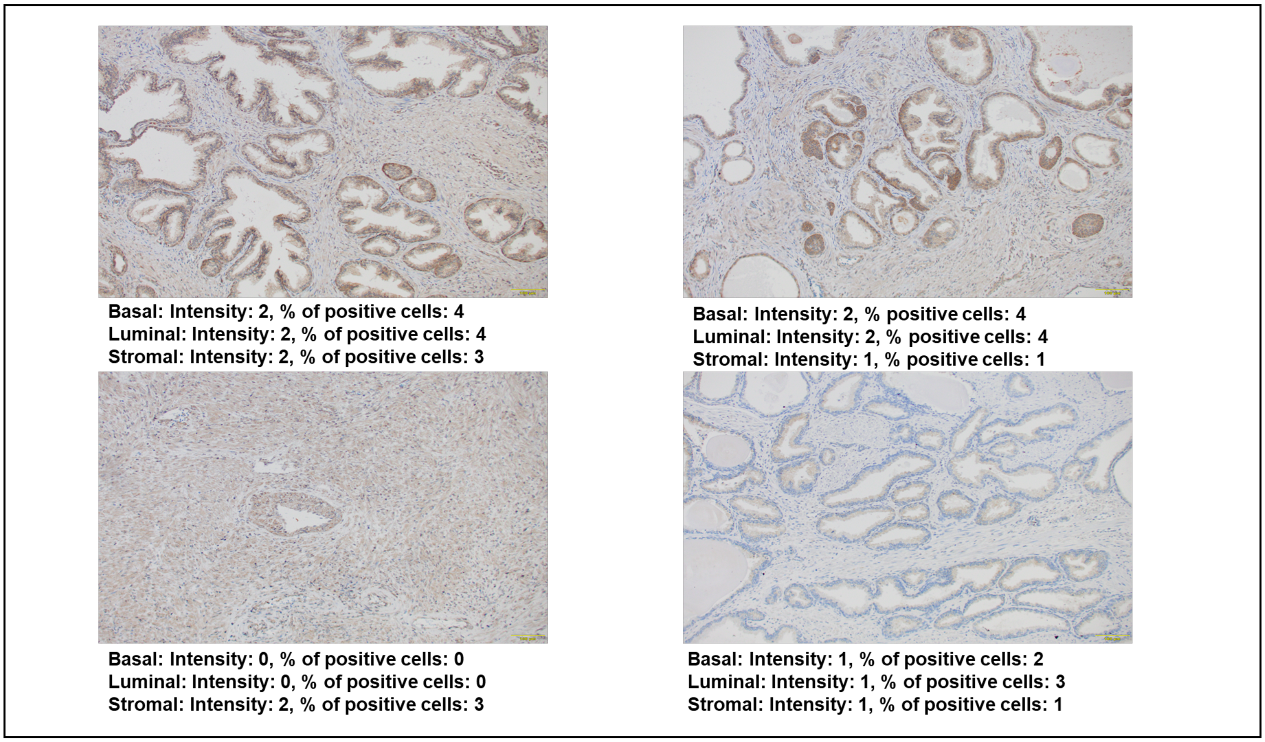


**Figure S2: Immunohistochemical staining for TIAM1 to illustrate Immunoreactive Scoring (IRS).** Representative IHC staining images of human benign prostate tissue samples, demonstrating varying staining intensities of staining and percentages of positive cells.

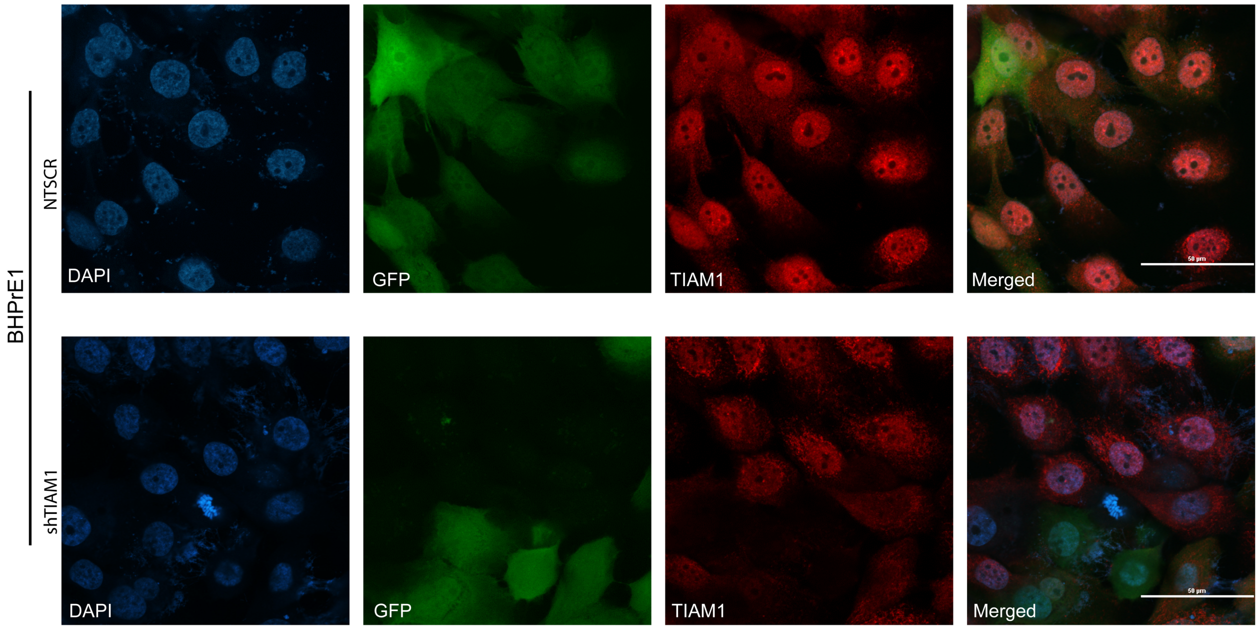


**Figure S3: Genetic knockdown of TIAM1 in BHPrE1 cells leads to decreased TIAM1 protein expression.** Immunofluorescence analysis showing decreased TIAM1 protein expression in BHPrE1$^{shTIAM1}$ cells compared to the control BHPrE1$^{NTSCR}$ cells.

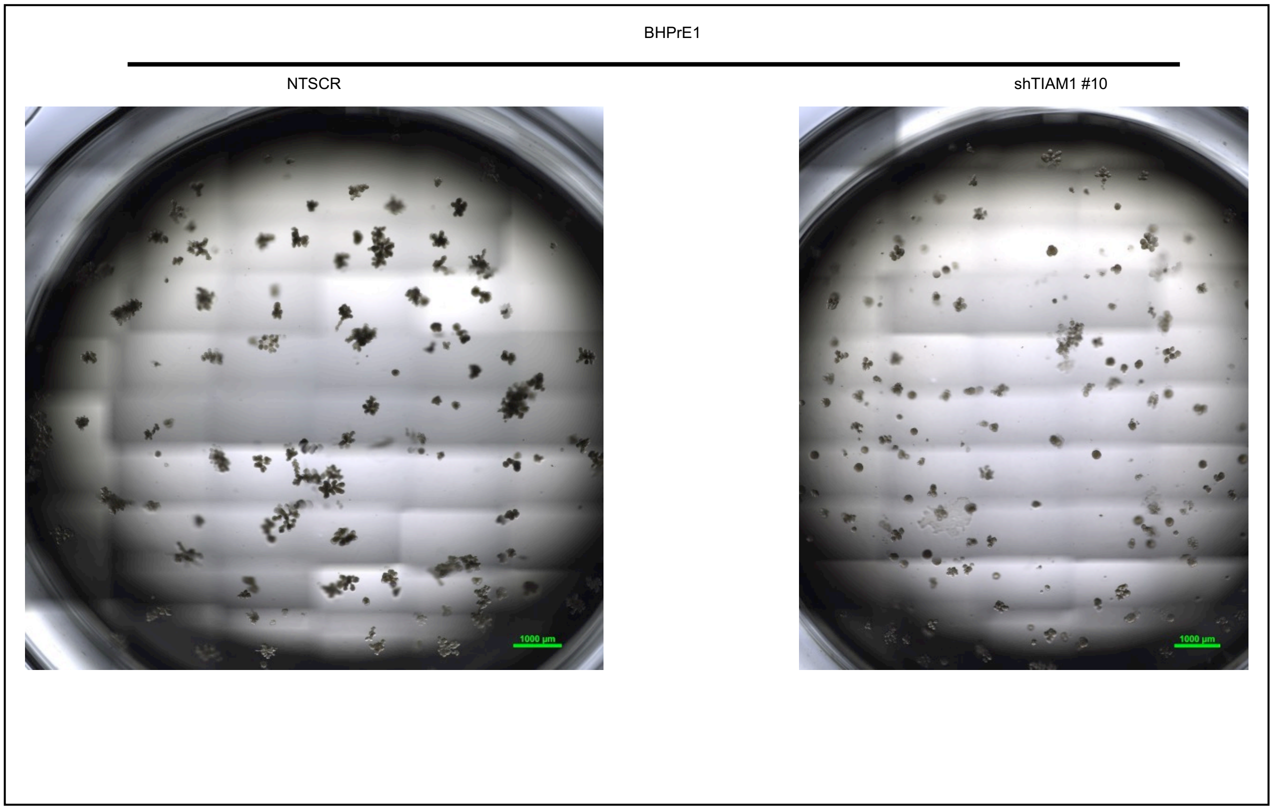


**Figure S4: Genetic knockdown of TIAM1 in BHPrE1 cells leads to a decrease in organoid branching phenotype.** Whole well images showing overall formation and branching morphogenesis of organoids formed from BHPrE1$^{shTIAM1}$ and control BHPrE1$^{NTSCR}$ cells in 3D cultures. The images were taken at 4X magnification across multiple microscopic planes and stitched together to form a larger image.

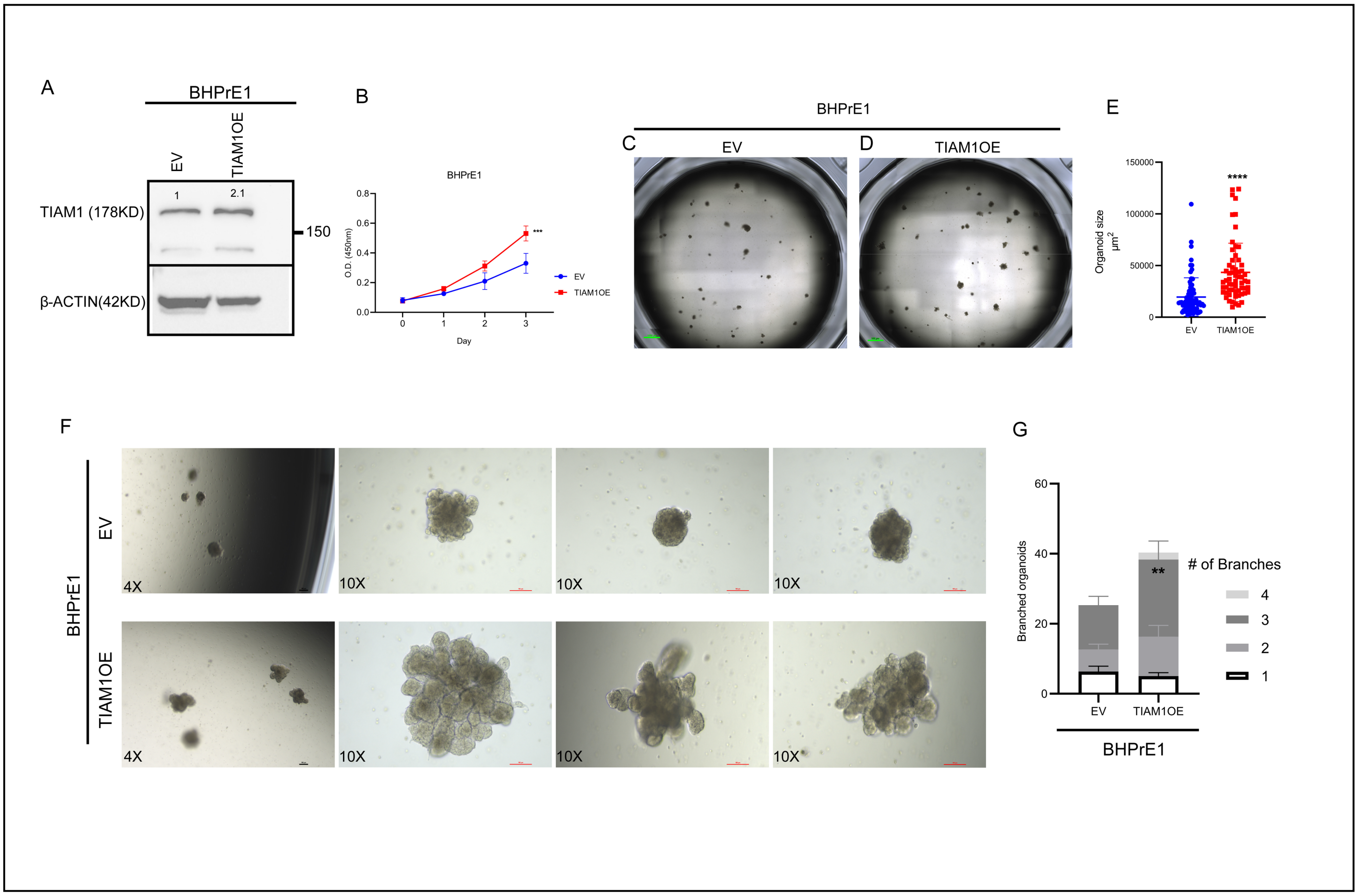


**Figure S5: Stable overexpression of TIAM1 increased organoid size and branching phenotype: BHPrE1 cells stably expressing TIAM1 were used for proliferation and organoid assays. A)** Western blot analysis showing increased TIAM1 protein expression in BHPrE1$^{TIAM1OE}$ compared to BHPrE1$^{EV}$ control cells. **B)** WST-1 assay showing enhanced cell proliferation in BHPrE1$^{TIAM1OE}$ compared to BHPrE1$^{EV}$ control cells (N=5, Mean ± SD). Unpaired two-tailed Student's t-test, ***; $P < 0.001$. Each experiment was performed in two independent replicates. **C, D)** Whole-well images of BHPrE1$^{TIAM1OE}$ and BHPrE1$^{EV}$ control cells taken on Day 12 (Scale bar: 1000 µM). **E)** Quantification of organoid size on Day 12, comparing BHPrE1$^{TIAM1OE}$ with BHPrE1$^{EV}$ control cells. Mean ± SD. Unpaired two-tailed Student's t-test. **F)** Representative images of organoids on Day 9 (4X) and Day 12 (10X) from BHPrE1$^{TIAM1OE}$ and BHPrE1$^{EV}$ control cells (Scale bar: 100 µM). **G)** Quantification of the number of branched organoids on Day 12 in BHPrE1$^{TIAM1OE}$ cells compared to BHPrE1$^{EV}$ control cells. N=3, Mean ± SD. Two-way ANOVA, **, $P < 0.01$. Each experiment was performed in two independent replicates.

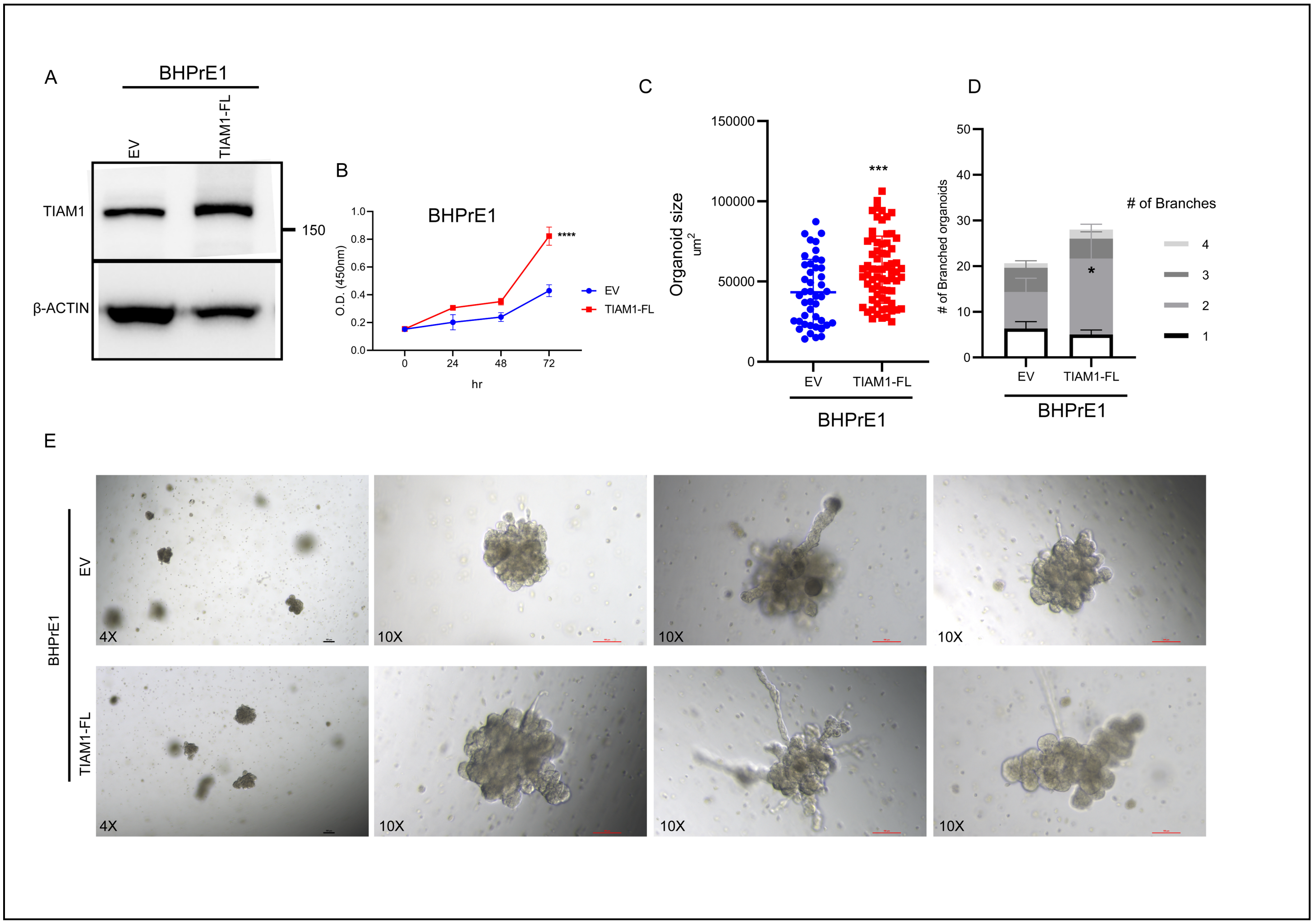


**Figure S6: Transient overexpression of TIAM1 increased organoid size and branching phenotype.** BHPrE1 cells transiently transfected with either an empty vector (EV) or TIAM1-full-length (TIAM1-FL) were used for proliferation and organoid assays. **A)** Western blot analysis showing increased TIAM1 protein expression in BHPrE1$^{TIAM1\text{-}FL}$ cells compared to BHPrE1$^{EV}$ control cells. **B)** WST-1 assay showing increased cell proliferation in BHPrE1$^{TIAM1\text{-}FL}$ compared to BHPrE1$^{EV}$ control cells. N=5, Mean ± SD. Unpaired two-tailed Student's t-test, ****; $P < 0.0001$. **C)** Quantification of organoid size on Day 12, comparing BHPrE1$^{TIAM1\text{-}FL}$ with BHPrE1$^{EV}$ control cells. Mean ± SD. Unpaired two-tailed Student's t-test. **D)** Quantification of the number of branched organoids on Day 12 in BHPrE1$^{TIAM1\text{-}FL}$ cells compared to BHPrE1$^{EV}$ control cells. N=3, Mean ± SD. Two-way ANOVA, *, $P < 0.05$. **E)** Representative images of organoids on Day 12 from BHPrE1$^{TIAM1\text{-}FL}$ and BHPrE1$^{EV}$ control cells (Scale bar: 100 μM). Each experiment was performed in two independent replicates.

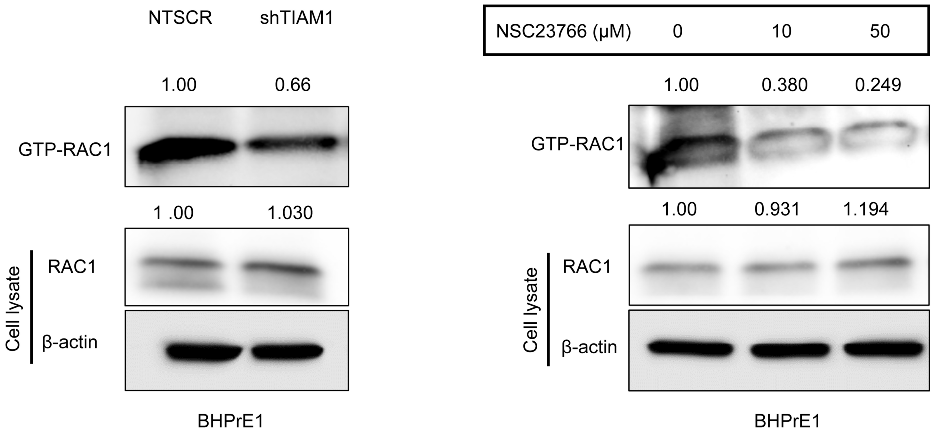


**Figure S7: Genetic knockdown of TIAM1 and NSC23766 treatment of BHPrE1 cells leads to reduced levels of active RAC1 (GTP-RAC1).** Active RAC1 (GTP-RAC1) pull-down assay in BHPrE1 cells. GTP-RAC1 was pulled down in **A)** BHPrE1$^{NTSCR}$, BHPrE1$^{shTIAM1}$ cells. **B)** BHPrE1 cells following 3 days of treatment with 10 μM and 50 μM concentrations of NSC23766. Cell lysates were prepared for all the cells and GTP-RAC1 was affinity precipitated using GST-PAK1-PBD/Glutathione resin, electrophoresed and detected using Rac1 mouse mAb. Western blot was also done to check the total RAC1 in all the cell lysates and quantified (represented in numbers). β-actin was used as an internal control.

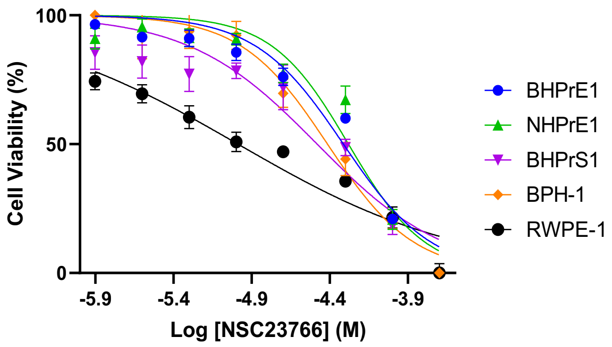


| Cell lines | IC50 (µM) | 95% CI (µM) |
|---|---|---|
| BHPrE1 | 48.6 | 41.4 to 56.5 |
| BPH-1 | 38.4 | 37.1 to 43.3 |
| NHPrE1 | 52.3 | 44.1 to 61.6 |
| RWPE-1 | 10.8 | 8.2 to 12.9 |
| BHPrS1 | 33 | 24.8 to 42.9 |

**Figure S8: The IC50 values for NSC23766 in human benign prostatic cell lines.** The WST-1 assay values obtained from NSC23766 (1.25 µM to 200 µM) treated cells for 72 hours were normalized to the values of untreated cells to determine the half-maximal inhibitory concentration (IC50). Table shows IC50 values with 95% confidence intervals (CI) values obtained for the NSC23766 dose response. (N=5 in each condition, Mean ± SEM).

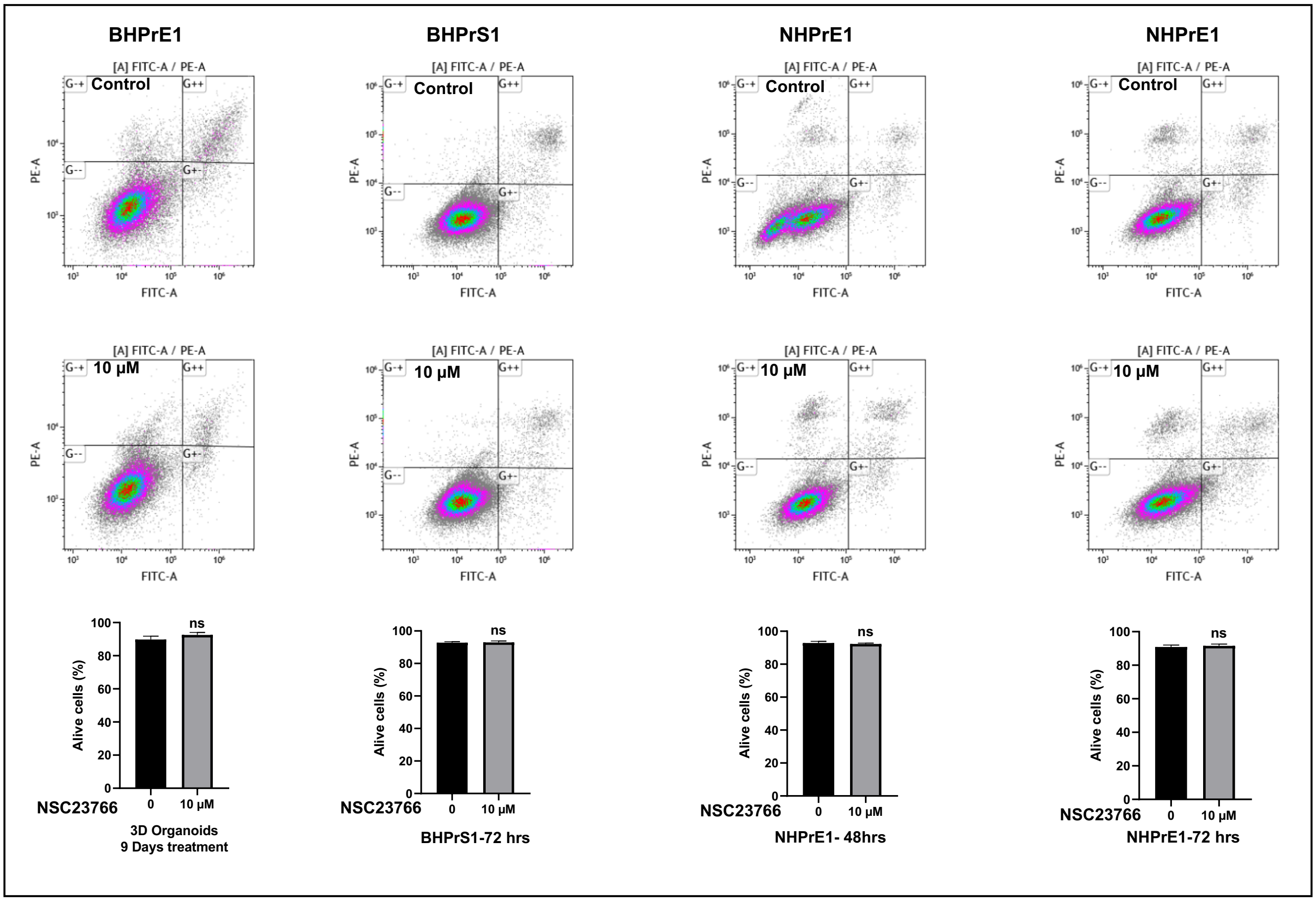


**Figure S9: NSC23766 does not induce significant apoptosis in prostate cells and organoids at 10 µM.** Apoptosis was evaluated in four different cell lines, including **(A)** BHPrE1 3D organoids, 48 hrs, **(B)** BHPrS1 cells in 2D culture, 48 hrs, NHPrE1 cells in 2D culture, 48 hrs **(C)** and 72 hrs **(D)**. Quantification of percentage of live cells showed there are no significant difference between NSC23766-treated and the control group across all cell lines and culture models.

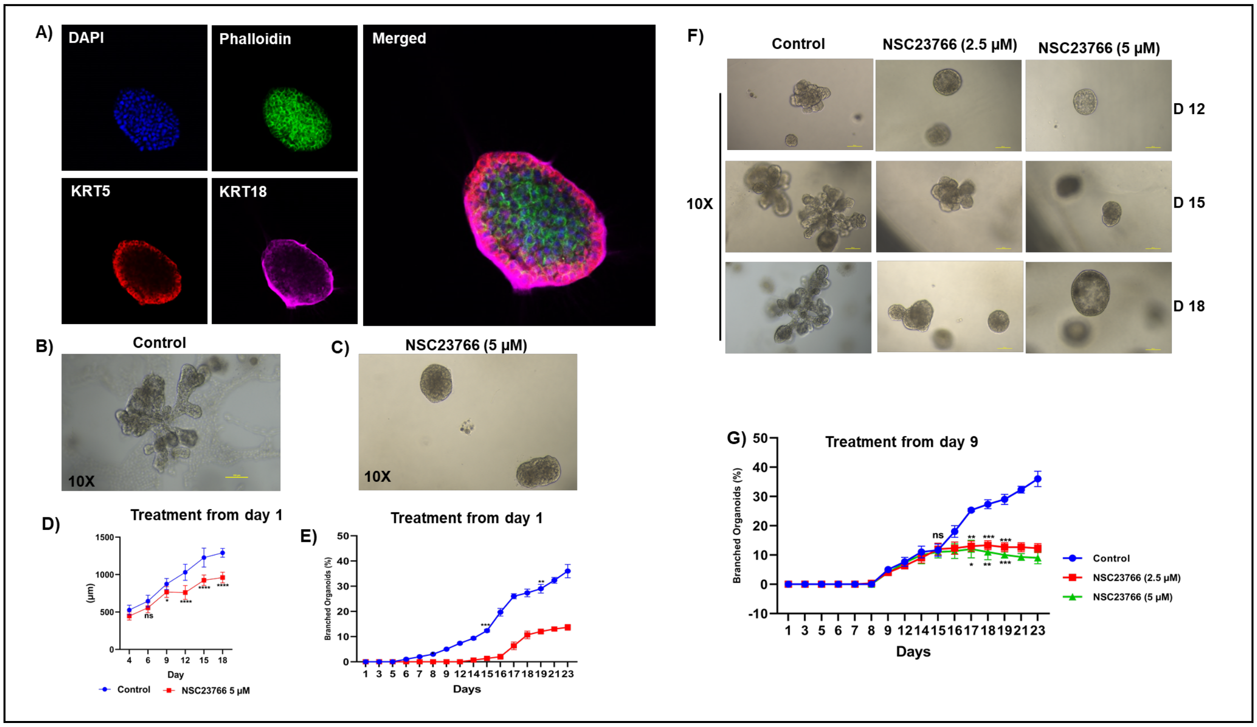


**Figure S10: NSC23766 caused a decrease in size and branching of RWPE-1 organoids in a dose-dependent manner. A)** Whole-mount Immunofluorescence staining of RWPE-1 organoids with basal cell marker (KRT5) and luminal cell marker (KRT8/18) on day 9. **B)** Representative images taken on day 12 of RWPE-1 organoids. **C)** Representative images taken on day 12 of RWPE-1 organoids with NSC23766 treatment starting on day 1 at 5 μM concentration. **D)** Quantification of the size of RWPE-1 organoids with NSC23766 treatment starting on day 1 at 5 μM concentrations compared to vehicle control. **E)** Quantification of the percentage of RWPE-1 branched organoids with NSC23766 treatment starting on day 1 at 5 μM concentrations compared to vehicle control. **F)** Representative images of RWPE-1 with NSC23766 treatment starting on day 1 at 2.5 μM and 5 μM concentrations compared to vehicle control. **G)** Quantification of the percentage of RWPE-1 branched organoids with NSC23766 treatment starting on day 1 at 2.5 μM and 5 μM concentrations compared to vehicle control. (N = 3, Mean ± SD, one-way ANOVA was performed, *, $P < 0.05$; **, $P < 0.01$; ***, $P < 0.001$; ****; $P < 0.0001$). Each experiment was performed in three independent replicates.

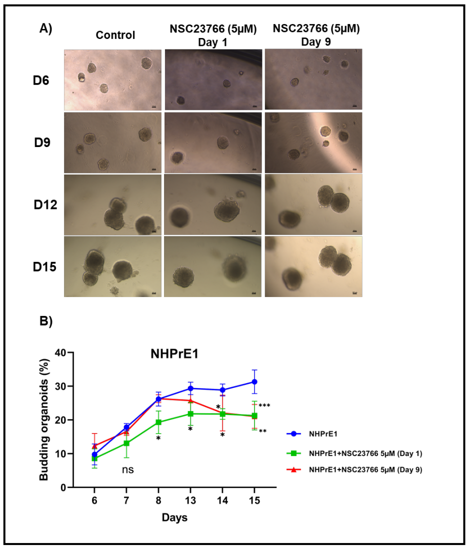


**Figure S11: NSC23766 caused a decrease in budding of NHPrE1 organoids. A)** Representative images taken on days 6, 9, 12 and 15 of NHPrE1 organoids treated with Vehicle control or with NSC23766 treatment on day 1 and Day 9. **B)** Quantification of the percentage of NHPrE1 budded organoids following treatment with NSC23766 on day 1 or day 9. (N=3, Mean ± SD, two-way ANOVA was performed, *, $P < 0.05$; **, $P < 0.01$; ***, $P < 0.001$). Each experiment was performed in two independent replicates.

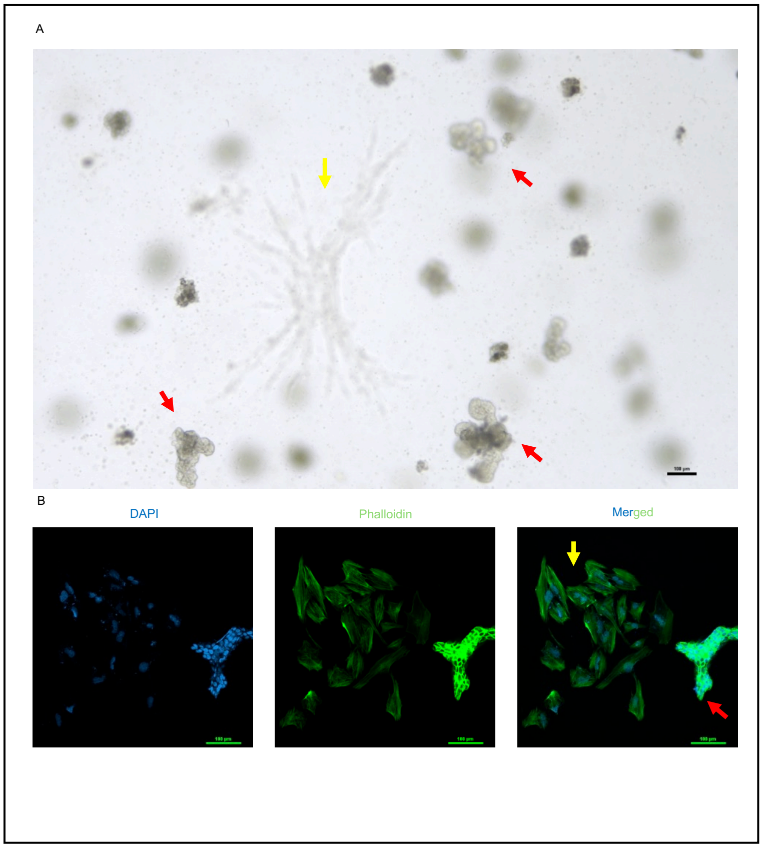


**Figure S12: Presence of BHPrS1 cells in co-culture with BPHrE1 organoids. A)** Magnified version of one of the images in 7A: bright-field image of BPHrS1 cells (yellow arrow) and BHPrE1 organoids (red arrows). **B)** Immunofluorescent image of BHPrS1 (yellow arrow) and BHPrE1 organoids (red arrows) in co-culture condition (Scale bar: 100 µm).

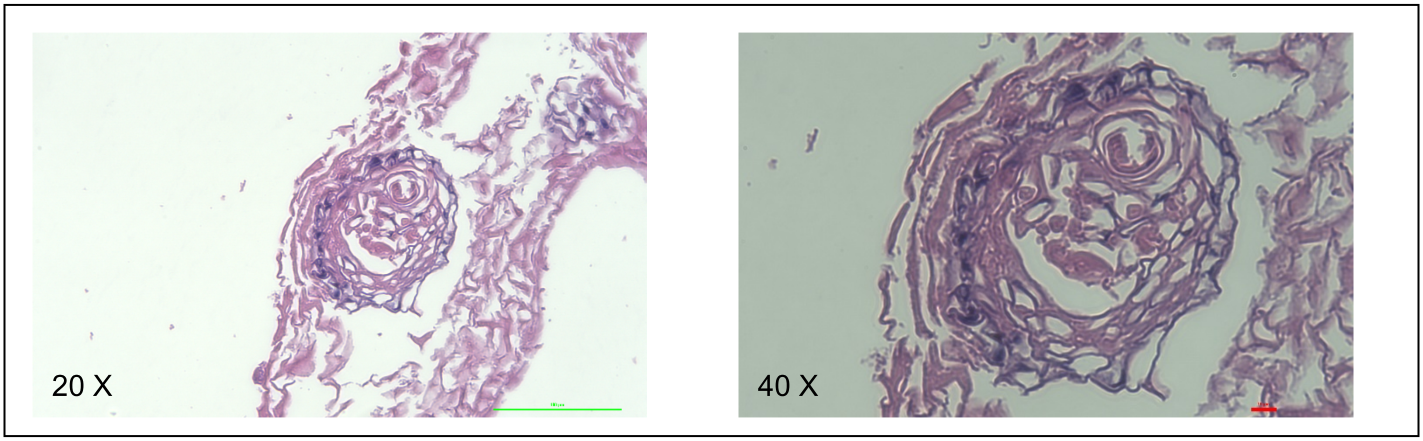


**Figure S13: H&E staining of PDOs**. Representative images in 20X, Scale bar: 100 µm and 40X, Scale bar: 10 µm.

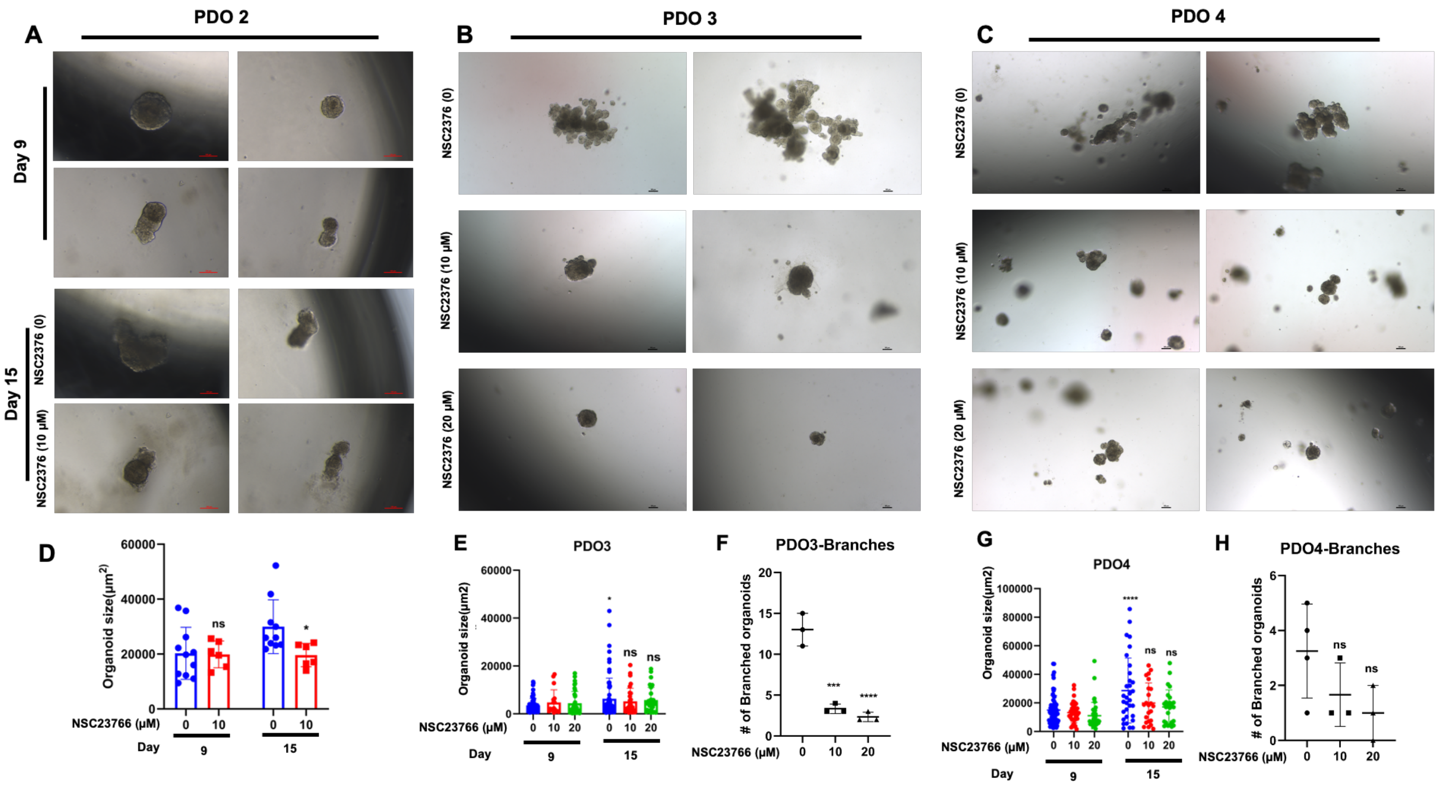


**Figure S14: NSC23766 exposure suppresses PDO growth.** Patient-derived organoid (PDO) cultures were exposed to NSC23766 (10 µM or 20 µM) and maintained in culture for 15 days. **A-C)** Representative images of PDOs 2, 3, and 4 treated with 10 µM or 20 µM NSC23766 compared to the vehicle control, captured at 4X magnification (Scale bar: 100 µm), on day 9 (before treatment) and day 15 (post-treatment). **E-H)** Quantification of organoid size for PDOs 2, 3, 4 on day 9 (before treatment) and day 15 (post-treatment). Paired two-tailed Student's t-test was performed to compare day 9 and day 15 values for each treatment group. *, $P < 0.05$; **, $P < 0.01$; ***, $P < 0.001$; ****, $P < 0.0001$.
