# Supplementary figures and images for: TIAM1 drives prostatic branching phenotype and is a potential therapeutic target for benign prostatic hyperplasia
Source: JCI Insight. 2025 May 20;10(12):e188062. doi: 10.1172/jci.insight.188062 (PMC12220968; doi:10.1172/jci.insight.188062)

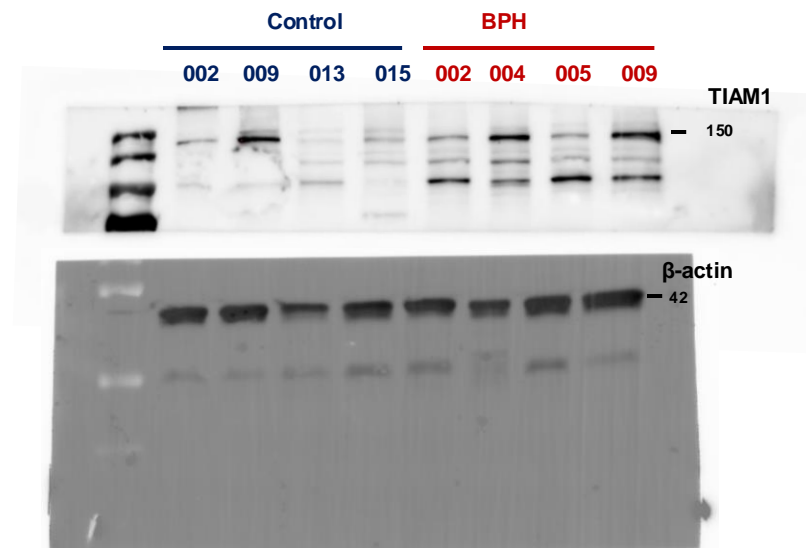

Full blot for figure 3 D

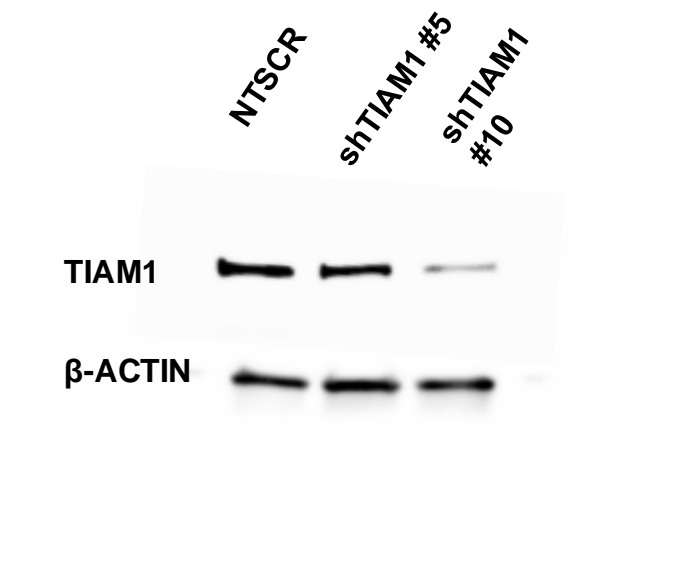

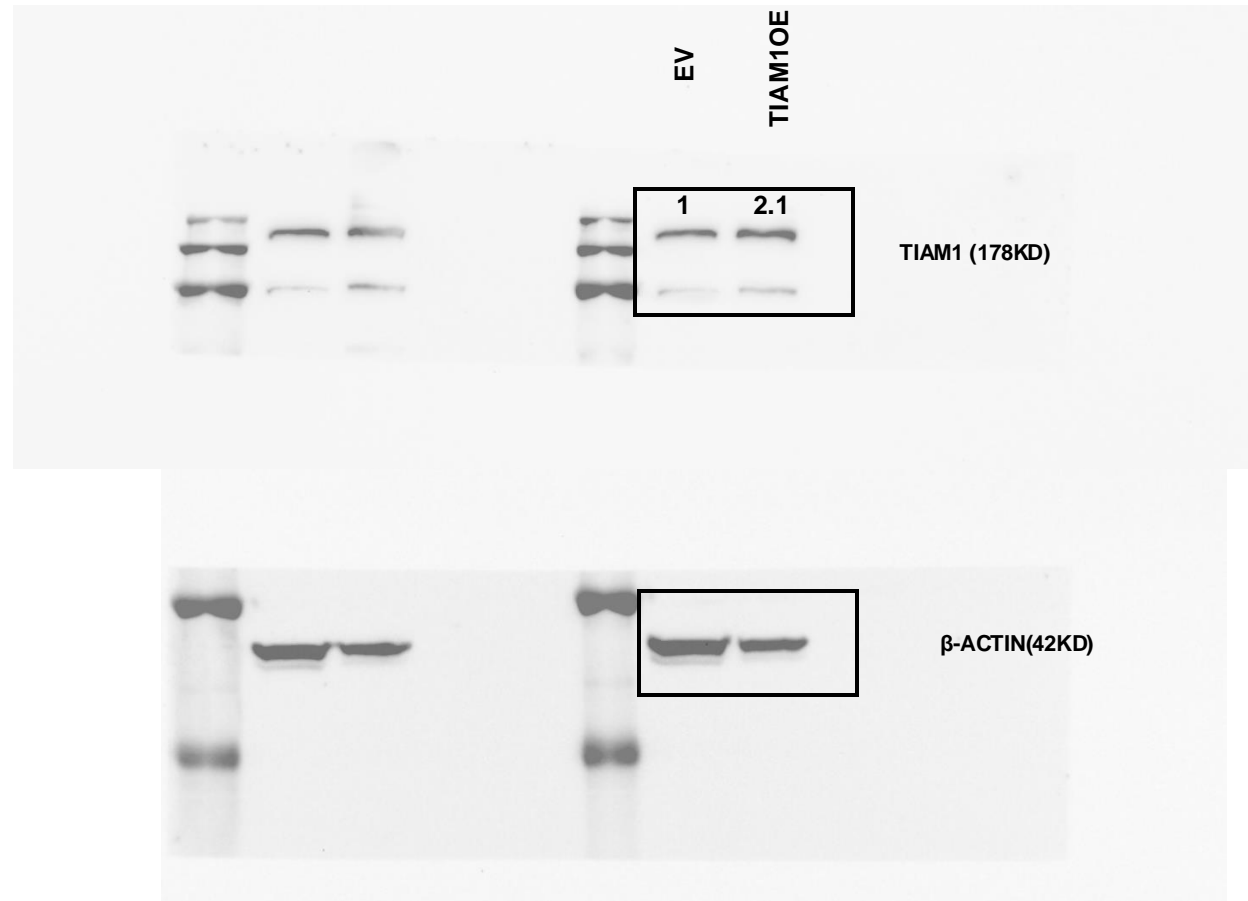

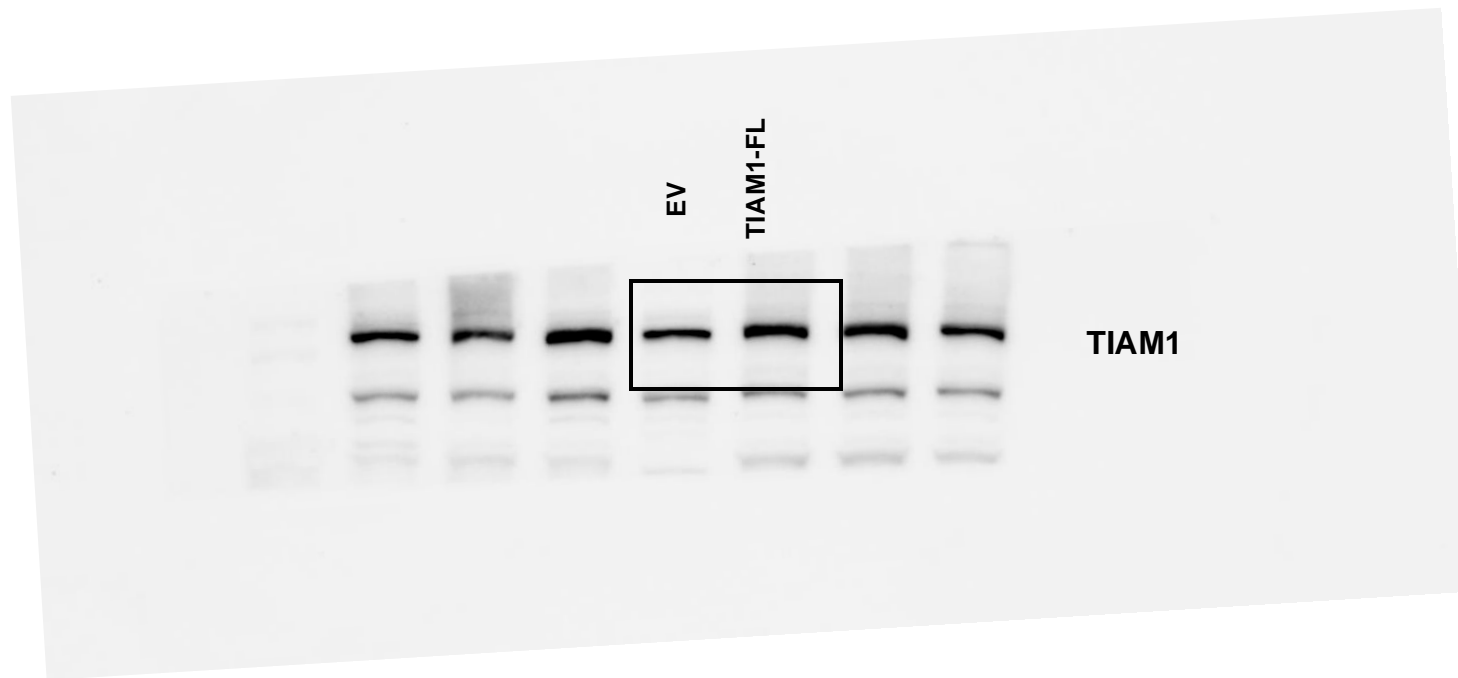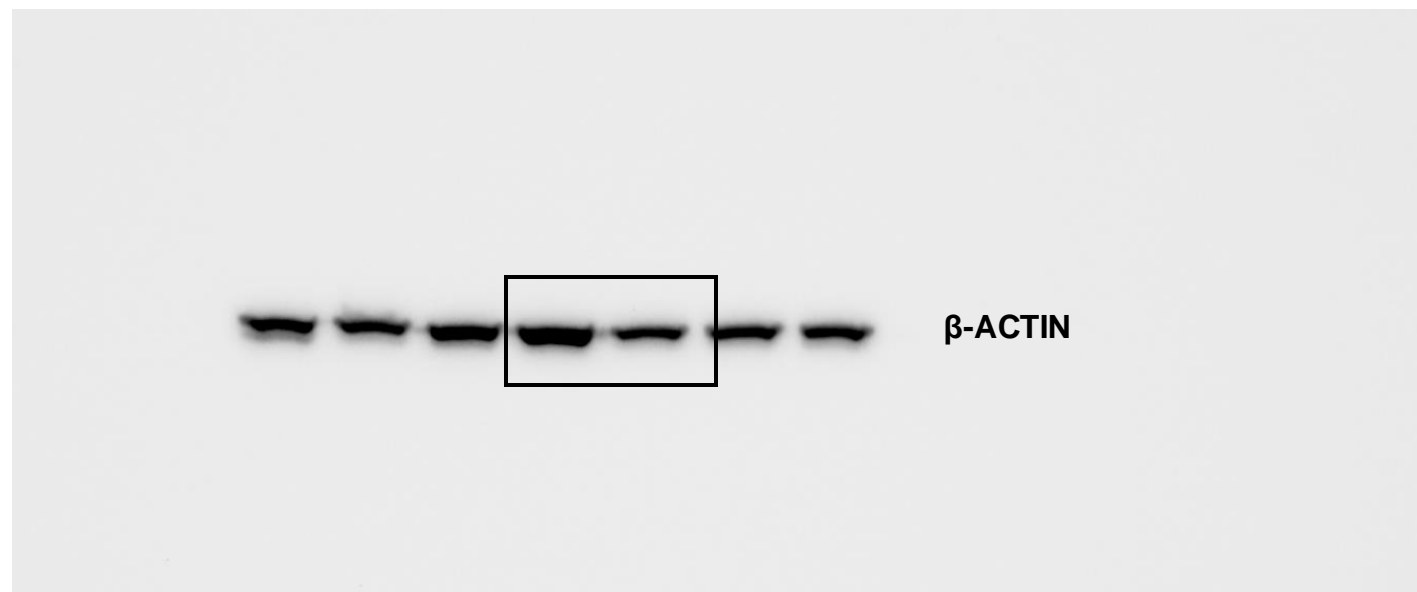

Full blot for figure S6

Supplement: Unedited blot and gel images [file jciinsight-10-188062-s142.pdf]
